# Supplementary material for: Antimicrobial Properties of Amino-Acid-Derived N-Heterocyclic Carbene Silver Complexes
Source: Pharmaceutics. 2022 Mar 30;14(4):748. doi: 10.3390/pharmaceutics14040748 (PMC9024828; doi:10.3390/pharmaceutics14040748)
Supplement: Supplementary file 1 [file pharmaceutics-14-00748-s001.zip › pharmaceutics-1621600-supplementary.pdf]

Antimicrobial properties of amino acid-derived N-heterocyclic carbene silver complexes

Adrián Sánchez, Carlos J. Carrasco, Francisco Montilla, Eleuterio Álvarez, Agustín Galindo, María Pérez-Aranda, Eloísa Pajuelo, and Ana Alcudia

SUPPLEMENTARY DATA

Table S1. Crystal data and structure refinement for **2a**.

Table S2. Selected structural parameters for **2a** (bond lengths [Å] and angles [°]).

Figure S1. Coordination 1D polymer formed by **2a** viewed along *a* axis.

Figure S2. 3D packing of **2a** viewed along *b* axis.

Figure S3. Non-classical C-H···O hydrogen bonds in the unit cell of **2a**. **Microbiological assays.**

Figure S4. Relative MDA quantification for complex **2b** (control is considered as 100%) for *E. coli* (in blue) and *P. aeruginosa* (in red) assays. Control ranges are 0-0.2 nMol/mg for *E.coli* and 0.1-0.27 nMol/mg for *P. aeruginosa*.

Figure S5. Relative MDA quantification for complex **2c** (control is considered as 100%) for *E. coli* (in blue) and *P. aeruginosa* (in red) assays. Control ranges are 0-0.2 nMol/mg for *E.coli* and 0.1-0.27 nMol/mg for *P. aeruginosa*.

Figure S6. Evaluation of biofilm formation of *E. coli* (top) and *P. aeruginosa* (bottom) exposed to complex **2b** (A: control, B: MIC, C: MBC).

Figure S7. Evaluation of biofilm formation of *E. coli* (top) and *P. aeruginosa* (bottom) exposed to complex **2c** (A: control, B: MIC, C: MBC).

Figure S8. Optimised structures of compounds [$Im^{Mes,R}$], **1**.

Figure S9. Optimised structures of compounds **1'**, carbene isomers of [$Im^{Mes,R}$].

Figure S10. HOMOs of compounds **1'**.

Figure S11. Optimised structures of {Ag[$NHC^{Mes,R}$]}$_2$ complexes, **2b$_c$-2d$_c$**.

Figure S12. Optimised structures of [$NHC^{Mes,R}$]$^-$ ligands.

Figure S13. Selected MOs of [$NHC^{Mes,R}$]$^-$ ligands.

Figure S14. ^{1}H-DOSY spectrum (500 MHz, 25 °C, Cl_3CD) of the mixture of **1a** and **2a**.

Figure S15. Determination of the maximum distance between the two farthest atoms in the optimised structures of compound [$Im^{Mes,H}$], **1a**, and complex {Ag[$NHC^{Mes,H}$]}$_2$, **2a**.

Table S3. Coordinates of the optimised compounds.

Table S1. Crystal data and structure refinement for **2a**.

Compound	**2a**
Formula	$C_{14}H_{15}AgN_2O_2$
M	351.15
T [K]	193(2)
Wavelength [Å]	0.71073
Crystal system	Monoclinic
Space group	$P2_1/c$
a [Å]	9.3237(12)
b [Å]	11.6432(13)
c [Å]	17.894(2)
$\alpha = \gamma$ [°]	90
β [°]	92.359(6)
V [Å^3]	1940.9(4)
Z	4
$D_{calcd.}$ [Mg·m^{-3}]	1.202
μ [mm^{-1}]	1.038
F(000)	704
Crystal size [mm^3]	0.250 x 0.100 x 0.050
θ range for data collection [º]	2.087 to 27.747
Index ranges	$-12\leq h\leq 12$, $-15\leq k\leq 15$, $-23\leq l\leq 23$
Reflections collected	43542
Independent reflections	4565 [R(int) = 0.1133]
Completeness to theta	= 25.242° 100.0 %
Absorption correction	Semi-empirical from equivalents
Max. and min. transmission	0.7461 and 0.4620
Refinement method	Full-matrix least-squares on F^2
Data / restraints / parameters	4565 / 18 / 175
Goodness-of-fit on F^2	1.099
Final R indices [I>2σ(I)]	R1 = 0.0955, wR2 = 0.2447
R indices (all data)	R1 = 0.1269, wR2 = 0.2582
Extinction coefficient	n/a
Largest diff. peak and hole [e·Å^{-3}]	2.903 and -1.693

Table S2. Selected structural parameters for **2a** (bond lengths [Å] and angles [°]).

Ag(1)-C(1)	2.046(8)	C(4)-C(9)	1.379(15)
Ag(1)-O(1)#1	2.100(6)	C(4)-C(5)	1.390(11)
O(1)-C(14)	1.245(11)	C(5)-C(6)	1.378(15)
O(2)-C(14)	1.187(12)	C(5)-C(10)	1.496(17)
N(1)-C(3)	1.362(12)	C(6)-C(7)	1.357(19)
N(1)-C(1)	1.364(10)	C(7)-C(8)	1.386(19)
N(1)-C(4)	1.444(11)	C(7)-C(11)	1.497(16)
N(2)-C(1)	1.348(11)	C(8)-C(9)	1.347(17)
N(2)-C(2)	1.393(12)	C(9)-C(12)	1.539(16)
N(2)-C(13)	1.449(11)	C(13)-C(14)	1.553(13)
C(2)-C(3)	1.337(14)		

C(1)-Ag(1)-O(1)#1	170.4(3)	C(6)-C(5)-C(4)	116.8(10)
C(14)-O(1)-Ag(1)#2	114.2(6)	C(6)-C(5)-C(10)	122.2(9)
C(3)-N(1)-C(1)	112.2(7)	C(4)-C(5)-C(10)	121.1(9)
C(3)-N(1)-C(4)	125.6(7)	C(7)-C(6)-C(5)	123.1(11)
C(1)-N(1)-C(4)	122.1(7)	C(6)-C(7)-C(8)	117.1(11)
C(1)-N(2)-C(2)	110.8(7)	C(6)-C(7)-C(11)	120.4(14)
C(1)-N(2)-C(13)	124.2(7)	C(8)-C(7)-C(11)	122.5(15)
C(2)-N(2)-C(13)	124.8(8)	C(9)-C(8)-C(7)	123.2(13)
N(2)-C(1)-N(1)	103.6(7)	C(8)-C(9)-C(4)	117.7(11)
N(2)-C(1)-Ag(1)	133.0(6)	C(8)-C(9)-C(12)	122.9(12)
N(1)-C(1)-Ag(1)	123.4(6)	C(4)-C(9)-C(12)	119.4(10)
C(3)-C(2)-N(2)	107.0(8)	N(2)-C(13)-C(14)	111.5(7)
C(2)-C(3)-N(1)	106.5(8)	O(2)-C(14)-O(1)	128.7(9)
C(9)-C(4)-C(5)	122.1(9)	O(2)-C(14)-C(13)	120.0(8)
C(9)-C(4)-N(1)	119.1(8)	O(1)-C(14)-C(13)	111.3(8)
C(5)-C(4)-N(1)	118.7(8)		

Symmetry transformations used to generate equivalent atoms: #1 -x+1,y-1/2,-z+1/2. #2 -x+1,y+1/2,-z+1/2.

Figure S1. Coordination 1D polymer formed by **2a** viewed along *a* axis.

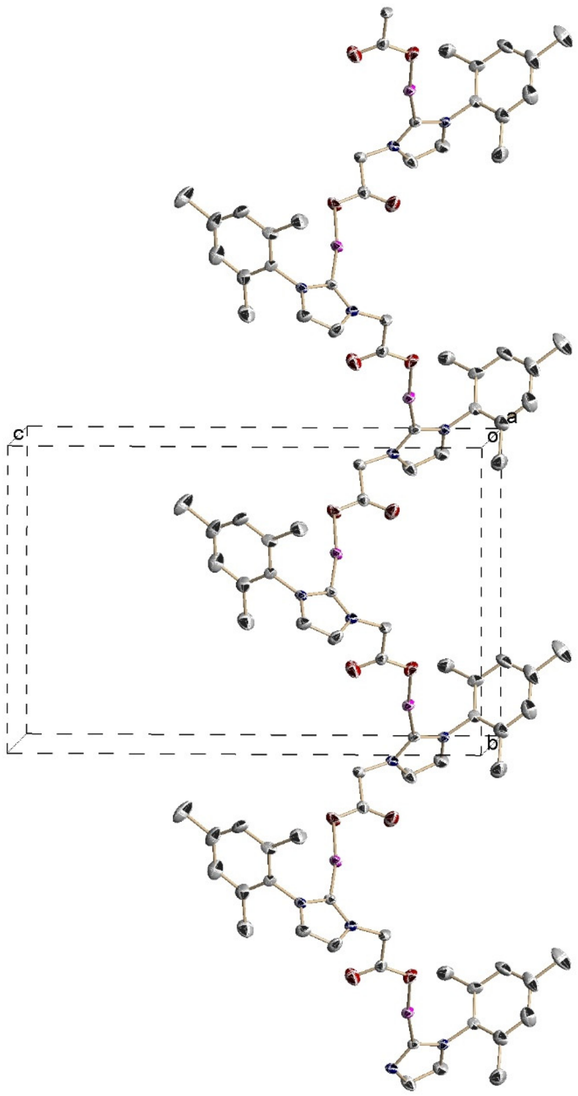

Figure S2. 3D packing of **2a** viewed along *b* axis.

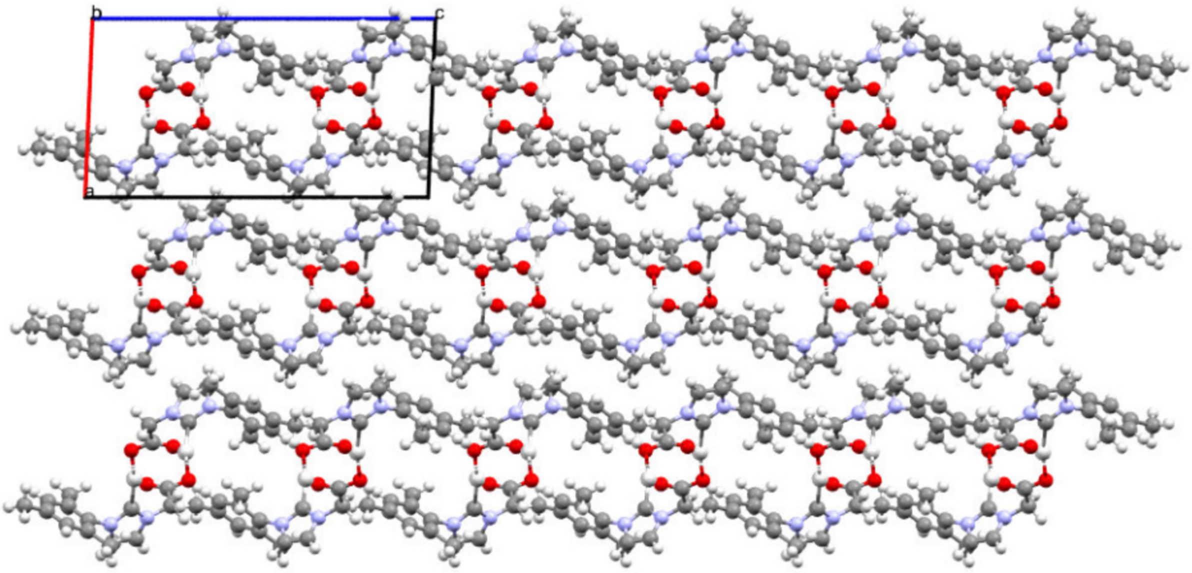

Figure S3. Non-classical C-H···O hydrogen bonds in the unit cell of **2a**.

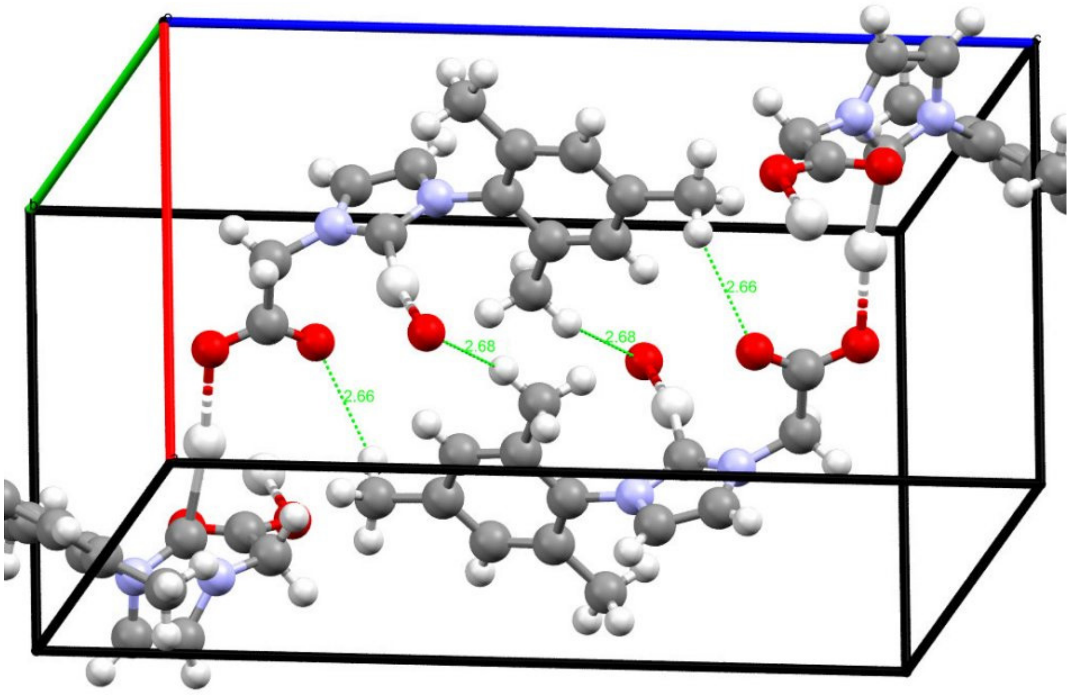

Figure S4. Relative MDA quantification for complex **2b** (control is considered as 100%) for *E. coli* (in blue) and *P. aeruginosa* (in red) assays. Control ranges are 0-0.2 nMol/mg for *E.coli* and 0.1-0.27 nMol/mg for *P. aeruginosa*.

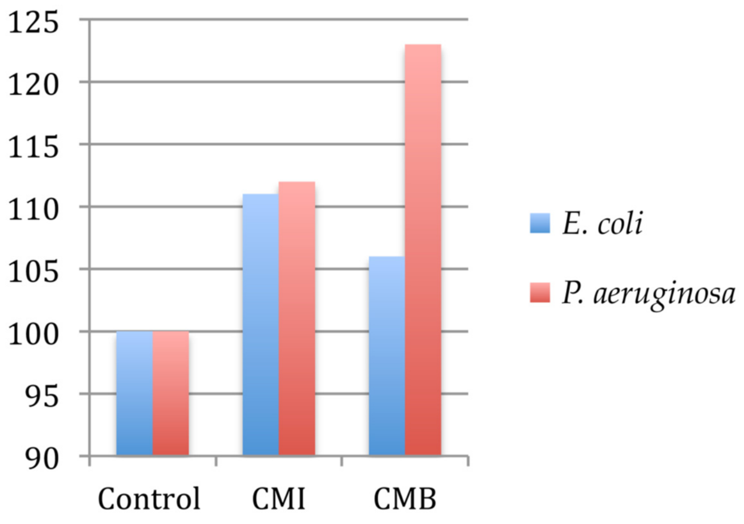

Figure S5. Relative MDA quantification for complex **2c** (control is considered as 100%) for *E. coli* (in blue) and *P. aeruginosa* (in red) assays. Control ranges are 0-0.2 nMol/mg for *E.coli* and 0.1-0.27 nMol/mg for *P. aeruginosa*.

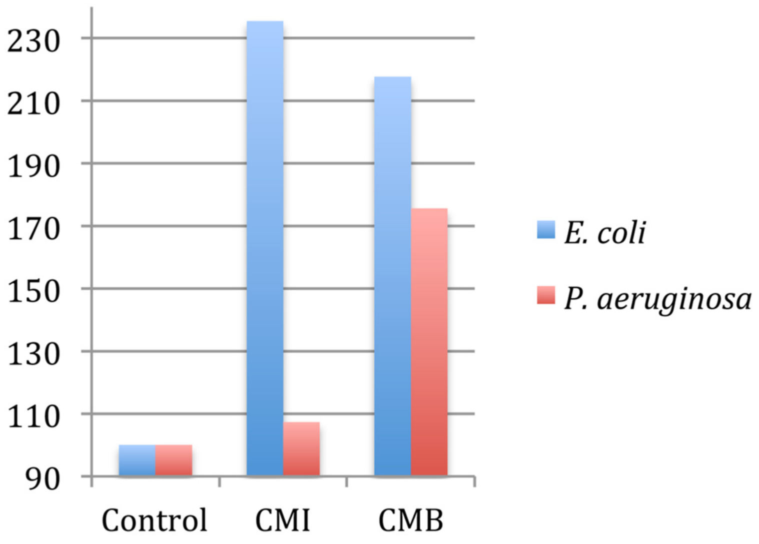

Figure S6. Evaluation of biofilm formation of *E. coli* (top) and *P. aeruginosa* (bottom) exposed to complex **2b** (A: control, B: MIC, C: MBC).

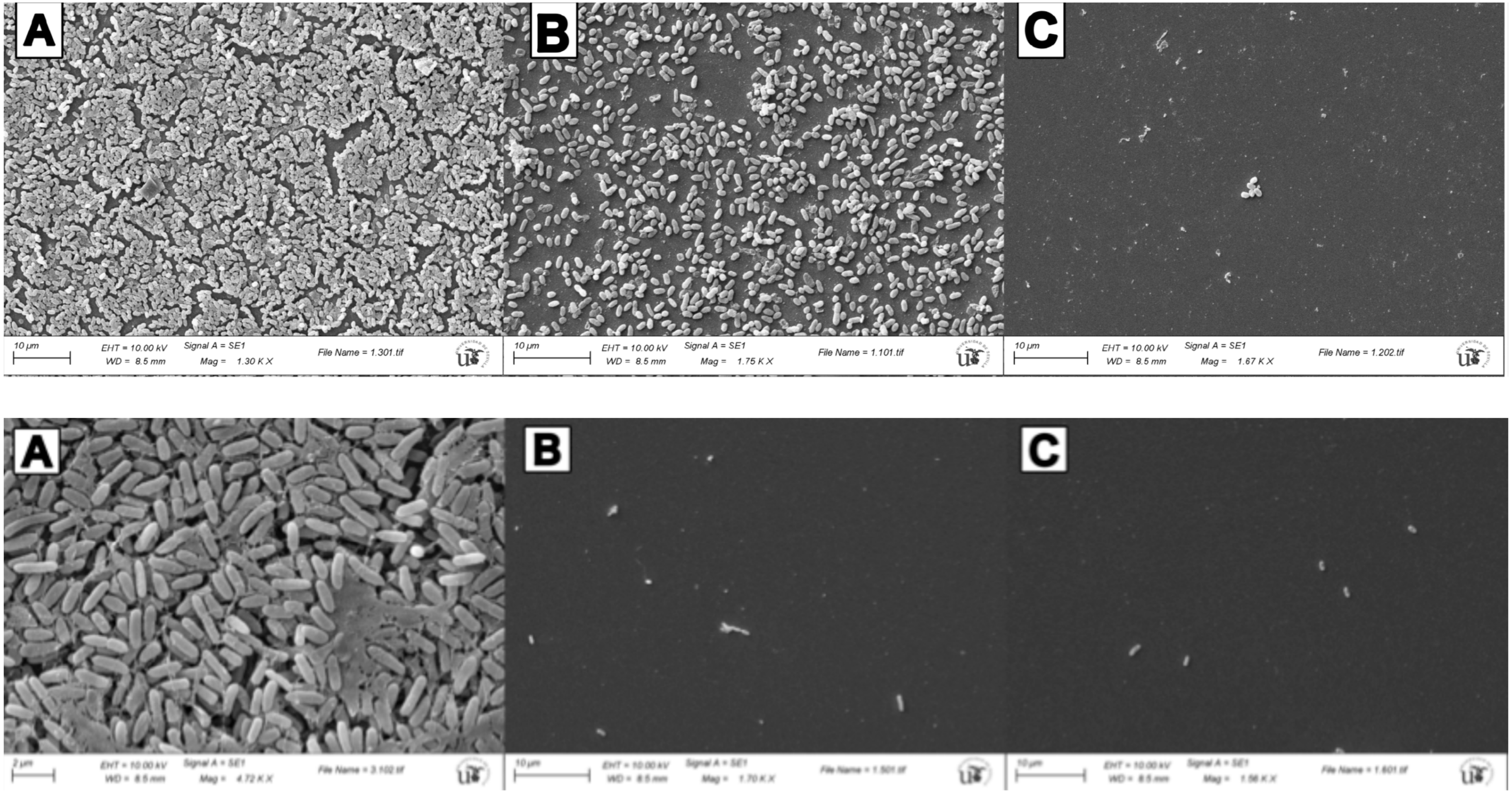

Figure S7. Evaluation of biofilm formation of *E. coli* (top) and *P. aeruginosa* (bottom) exposed to complex **2c** (A: control, B: MIC, C: MBC).

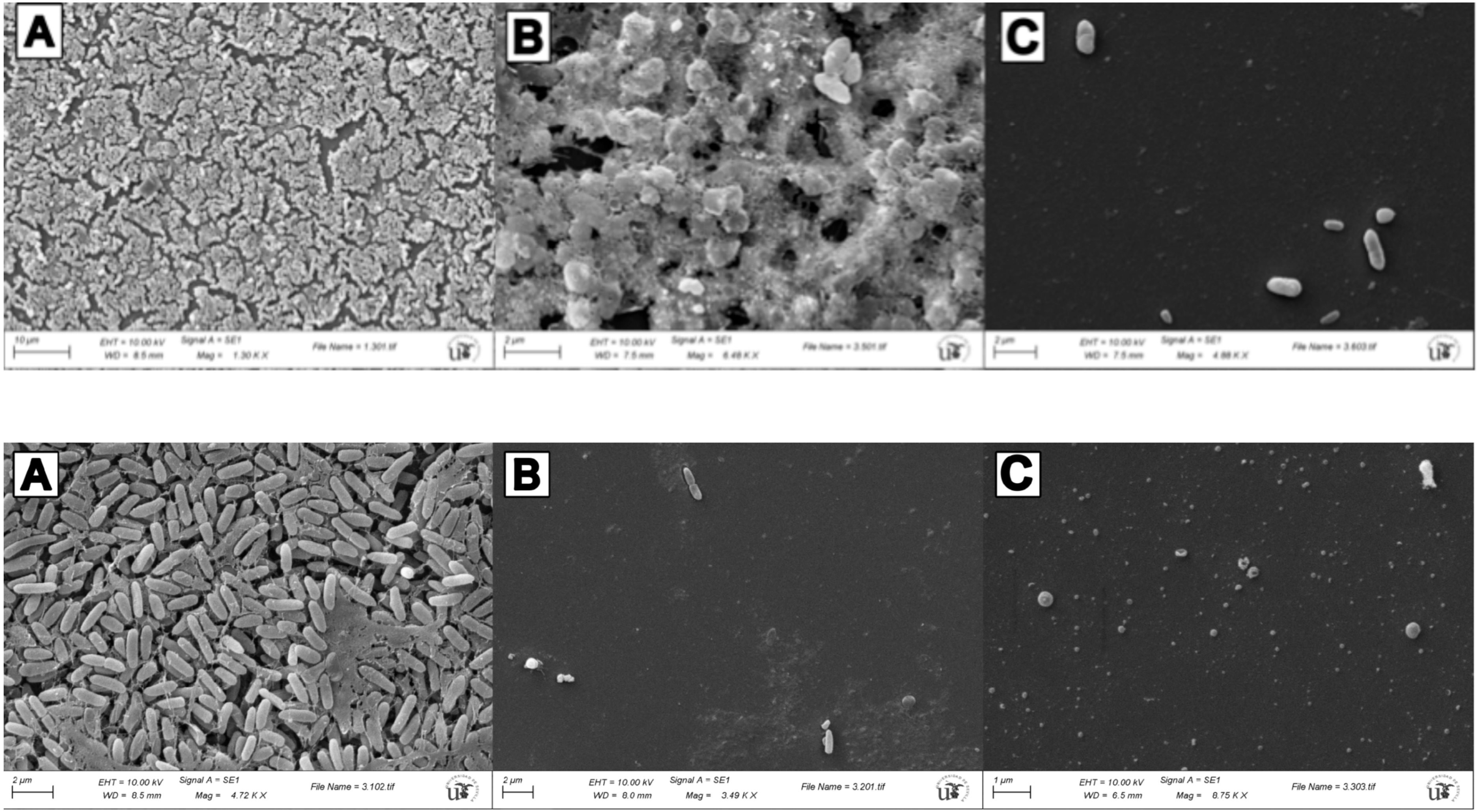

Figure S8. Optimised structures of compounds [Im^{Mes,R}], **1**.

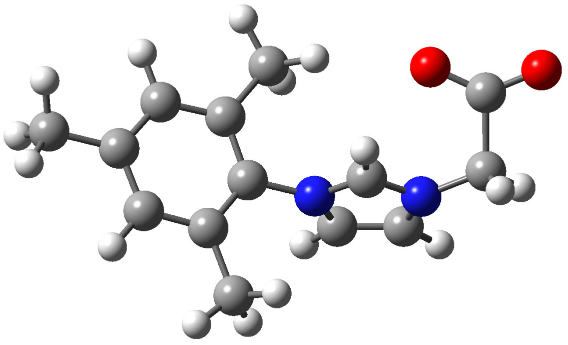

[Im^{Mes,H}]

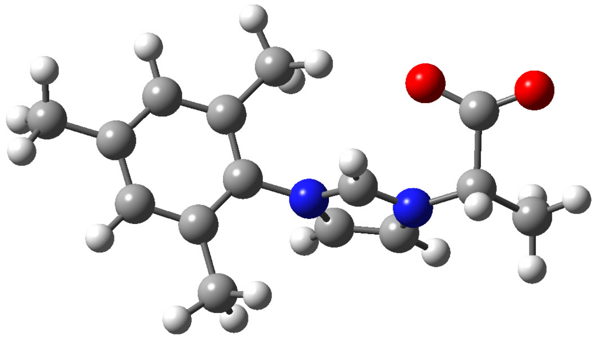

[Im^{Mes,Me}]

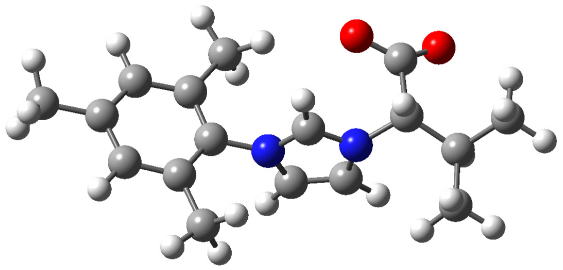

[Im^{Mes,iPr}]

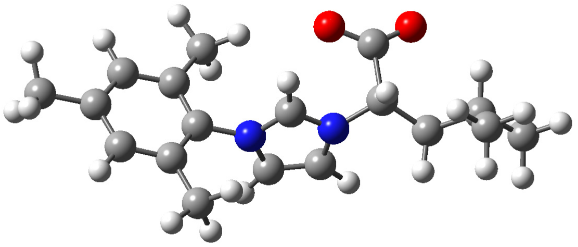

[Im^{Mes,iBu}]

Figure S9. Optimised structures of compounds **1'**, carbene isomers of [Im^{Mes,R}].

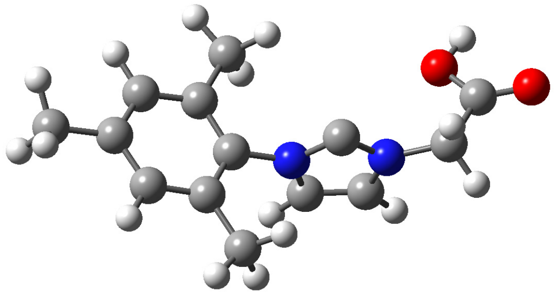

R = H

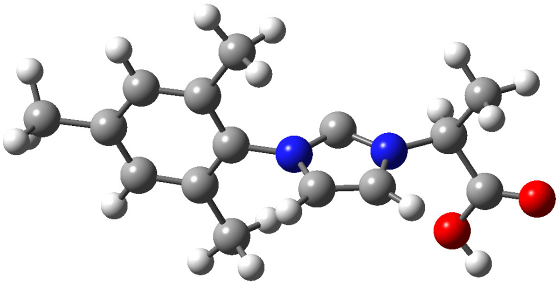

R = Me

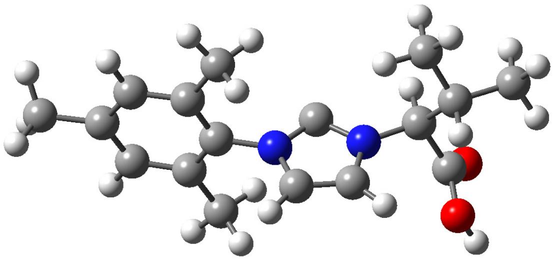

R = ^{i}Pr

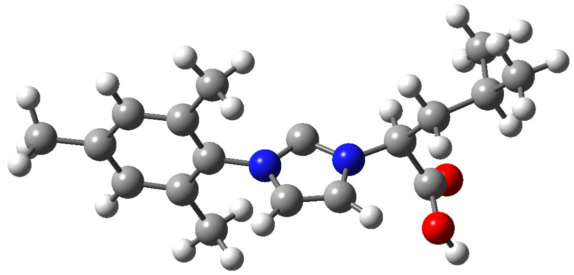

R = ^{i}Bu

Figure S10. HOMOs of compounds **1'**.

R = H

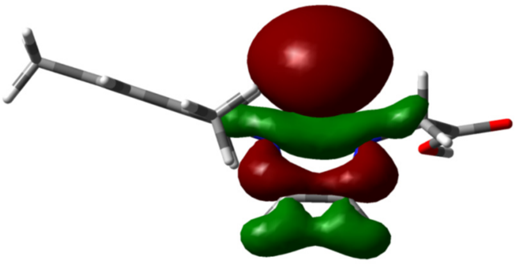

R = Me

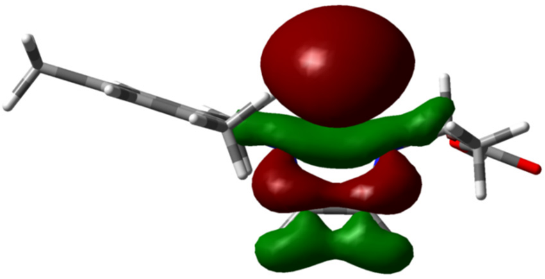

R = ^{i}Pr

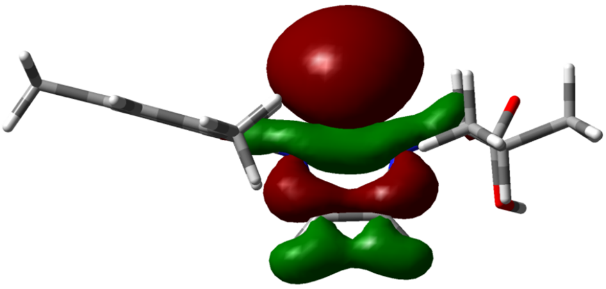

R = ^{i}Bu

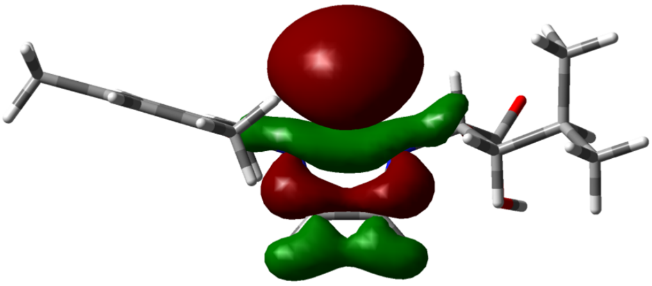

Figure S11. Optimised structures of $\{Ag[NHC^{Mes,R}]\}_2$ complexes, **$2b_c$-$2d_c$**.

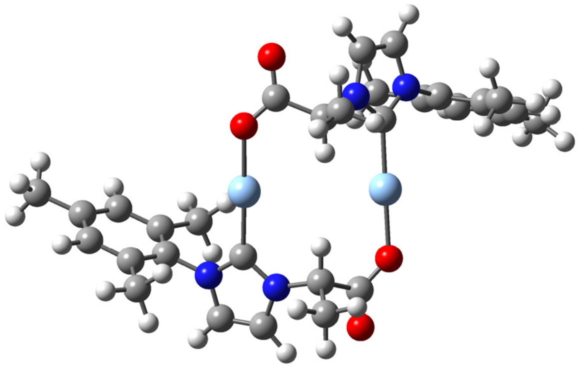

$\{Ag[NHC^{Mes,Me}]\}_2$, **$2b_c$**

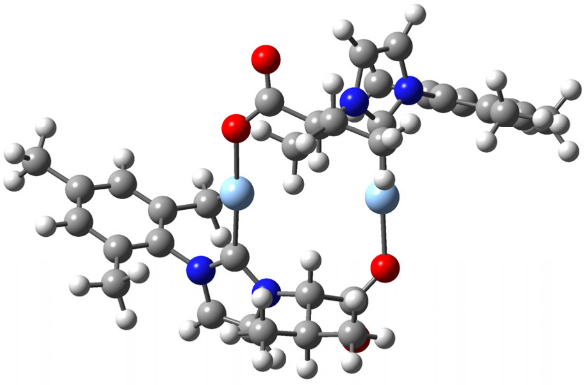

$\{Ag[NHC^{Mes,iPr}]\}_2$, **$2c_c$**

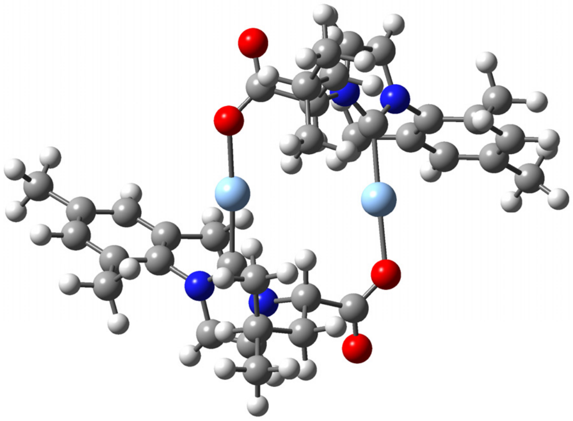

$\{Ag[NHC^{Mes,iBu}]\}_2$, **$2d_c$**

Figure S12. Optimised structures of $[NHC^{Mes,R}]^-$ ligands.

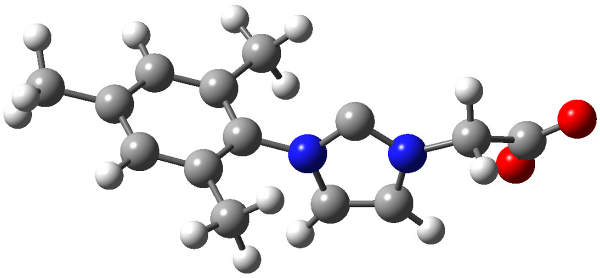

$[NHC^{Mes,H}]^-$

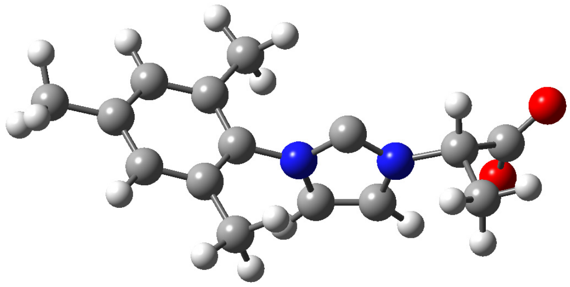

$[NHC^{Mes,Me}]^-$

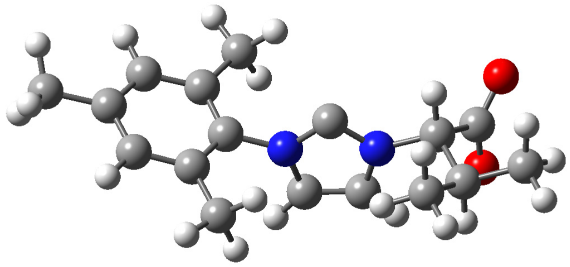

$[NHC^{Mes,iPr}]^-$

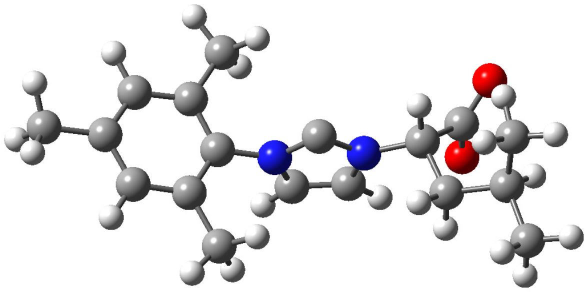

$[NHC^{Mes,iBu}]^-$

Figure S13. Selected MOs of $[NHC^{Mes,R}]^-$ ligands.

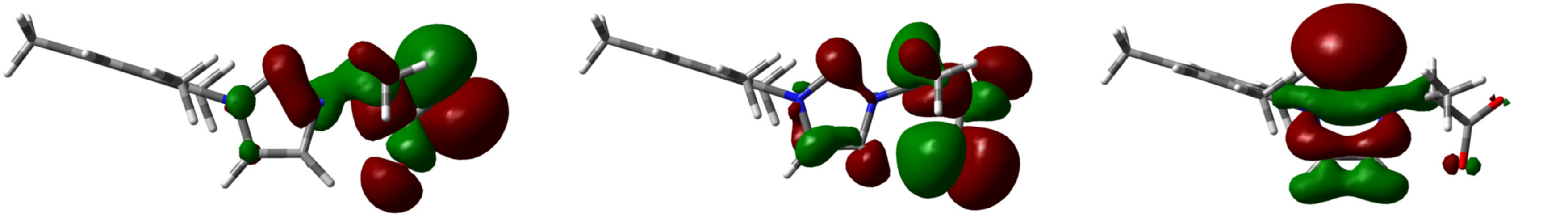

$[NHC^{Mes,Me}]^-$: HOMO HOMO-1 HOMO-3

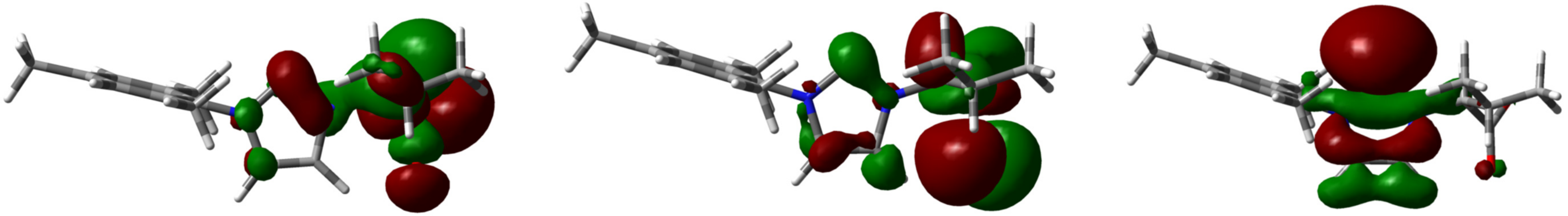

$[NHC^{Mes,iPr}]^-$: HOMO HOMO-1 HOMO-3

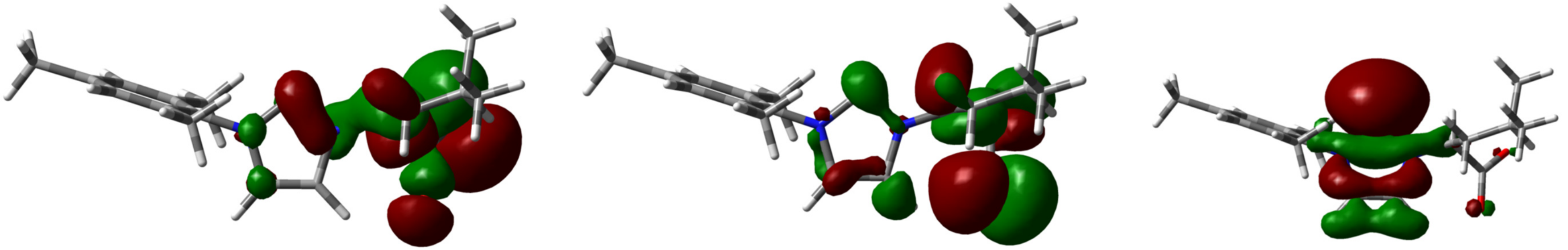

$[NHC^{Mes,iBu}]^-$: HOMO HOMO-1 HOMO-3

Figure S14. ^{1}H-DOSY spectrum (500 MHz, 300 K, Cl_3CD). Pulse program: ledbpgp2s, $\delta = 0.9$ ms, $\Delta = 120$ ms, LED = 5 ms, recycle delay: 5 s, 16 scans, time domain: 38k x 24 points, measurement time: 55 min, apodization: exponential broadening (0.3 Hz), spectrum: 32 k x 64 points.

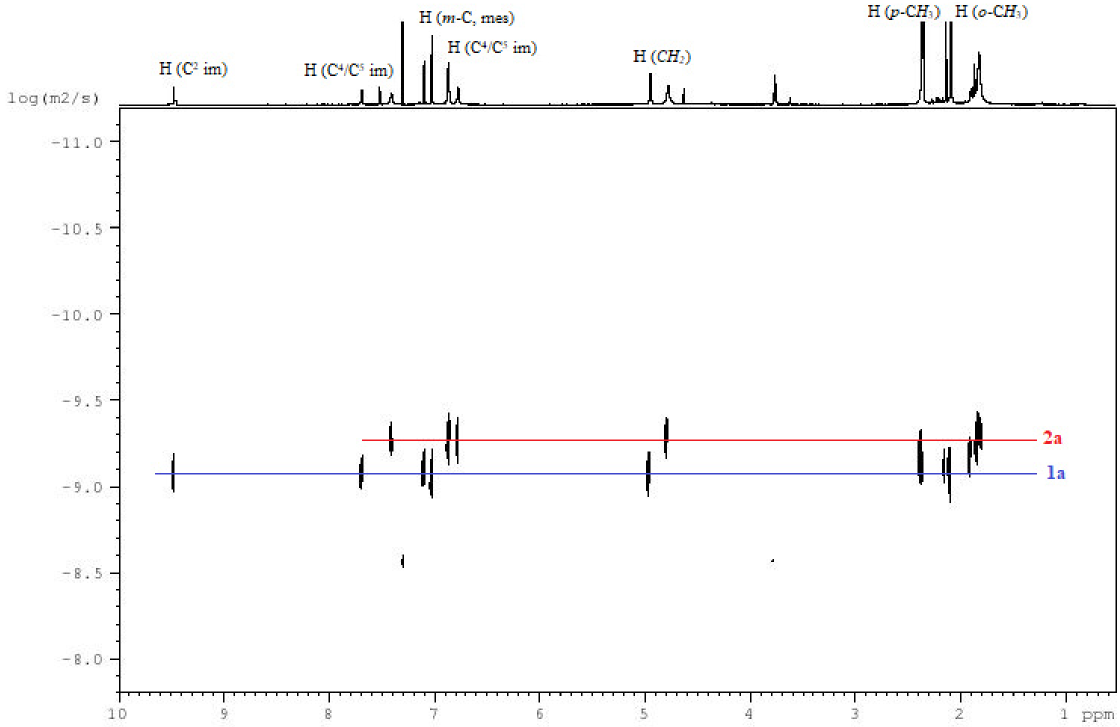

Figure S15. Determination of the maximum distance between the two farthest atoms in the optimised structures of compound [Im^{Mes,H}], **1a**, and complex {Ag[NHC^{Mes,H}]}$_2$, **2a**

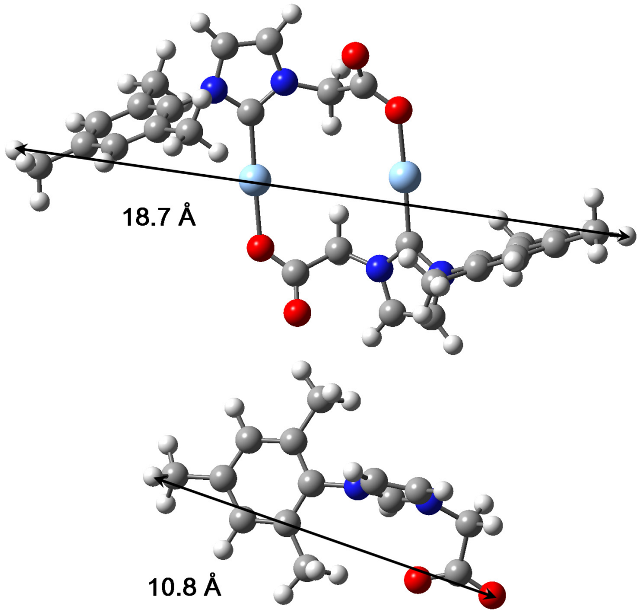

Table S3. Coordinates of the optimised compounds.

*Compounds [Im^{Mes,R}], **1**.*

[Im^{Mes,H}]

O	5.00639200	-1.34079900	-0.65947100
N	2.34501500	0.98106500	0.03314400
N	0.19840900	0.75119000	0.34068300
C	1.22388800	0.47488100	-0.48187000
C	0.69853000	1.43220400	1.44308700
H	0.06231500	1.74414700	2.25238200
C	2.03653900	1.57059200	1.24692700
H	2.79299200	2.02709100	1.86107000
C	3.67547800	0.59554200	-0.44934400
H	3.83328400	1.00374400	-1.44851900
C	3.83074400	-0.99728400	-0.53110600
O	2.75553200	-1.63484800	-0.48889500
C	-1.15585500	0.28057200	0.15381700
C	-2.11336100	1.15980400	-0.36689500
C	-1.44611100	-1.05078400	0.49090300
C	-3.40918700	0.67079300	-0.54346400
C	-2.75753900	-1.48585400	0.28736000
C	-3.74997700	-0.64499800	-0.22204900
H	-4.16589700	1.33315100	-0.95129800
H	-3.00571500	-2.51319700	0.53383200
C	-0.39954300	-1.97743500	1.06075400
H	0.49953600	-2.04215300	0.44064600
H	-0.07395400	-1.63714400	2.04935300
H	-0.80814200	-2.98194600	1.17572700
C	-1.76719900	2.58064500	-0.74202500
H	-1.44985700	3.16526000	0.12635300
H	-0.95098400	2.61844100	-1.46917600
H	-2.63080300	3.08045300	-1.18196100
C	-5.16255700	-1.14482600	-0.40088800
H	-5.71234900	-1.10070000	0.54534700
H	-5.71038100	-0.54291900	-1.12855900
H	-5.17637000	-2.18395400	-0.73749800
H	1.15441000	-0.08871400	-1.39310000
H	4.41944700	1.01473800	0.22429700

[Im^{Mes,Me}]

O	4.58137900	-1.71414500	-0.72233400
N	2.11001200	0.81132900	-0.02392400
N	-0.04181300	0.67441900	0.31995400
C	0.95776900	0.35190800	-0.51635100
C	0.50473500	1.33942300	1.40947900
H	-0.10413100	1.68628300	2.22555500
C	1.84421100	1.42079700	1.19172700
H	2.62013300	1.85588800	1.79417600
C	3.41411800	0.35588400	-0.55328000
H	3.44706700	0.66112800	-1.60265700
C	3.44531000	-1.26149800	-0.56996700
O	2.32602500	-1.81216700	-0.47456300
C	4.58607600	0.96971200	0.19379100
H	4.59787800	0.66609800	1.24411500
C	-1.42002100	0.27274600	0.15023800
C	-2.33440600	1.19481500	-0.37437500
C	-1.77768700	-1.03713300	0.50593000
C	-3.65554800	0.77285500	-0.53626100
C	-3.11170200	-1.40493300	0.31626100
C	-4.06238200	-0.51929700	-0.19657000
H	-4.37936500	1.46911600	-0.94740700
H	-3.41166600	-2.41486600	0.57675800
C	-0.77785300	-2.01054400	1.08125300
H	0.11761400	-2.12049600	0.46256900
H	-0.43724600	-1.68068500	2.06839200
H	-1.23522700	-2.99330000	1.20160600

C	-1.91716200	2.59115300	-0.76853700
H	-1.56405300	3.16814900	0.09107400
H	-1.10459600	2.57785100	-1.50060100
H	-2.75628800	3.13031900	-1.20949400
C	-5.50057700	-0.94629600	-0.35981000
H	-6.03926500	-0.86705200	0.59053800
H	-6.02275800	-0.32195000	-1.08746400
H	-5.57136400	-1.98569500	-0.68844000
H	0.84301100	-0.19818700	-1.43087600
H	4.59886200	2.06285900	0.13167900
H	5.49548000	0.57725100	-0.25771400

[Im^{Mes,iPr}]

O	3.72706500	-2.20629300	0.28954500
N	1.62577500	0.46165800	0.09134000
N	-0.53406100	0.45776000	0.41406800
C	0.44261000	0.24887300	-0.48379400
C	0.06087200	0.78965800	1.62383100
H	-0.52266800	0.99407200	2.50413000
C	1.40487500	0.78339400	1.42110200
H	2.21371300	0.95866100	2.10690800
C	2.87456600	-0.06752300	-0.50344600
H	2.77488700	0.09399900	-1.58087600
C	2.76500000	-1.67253900	-0.26668800
O	1.67784100	-2.12687600	-0.68618900
C	4.13662900	0.64419600	-0.00363900
H	4.29199600	0.36334700	1.04443500
C	5.35343600	0.13504300	-0.79386600
H	6.26430600	0.61944600	-0.43094300
H	5.45952300	-0.94257600	-0.68304200
H	5.25190800	0.37702800	-1.85843100
C	4.04355400	2.17552400	-0.12220600
H	3.90696700	2.47574100	-1.16696000
H	3.22118800	2.60577400	0.45525100
H	4.96926500	2.63795800	0.23162500
C	-1.94110900	0.23014800	0.17223000
C	-2.75632500	1.31939700	-0.16619200
C	-2.42516700	-1.08189800	0.27383900
C	-4.10678000	1.06422400	-0.40361000
C	-3.78655700	-1.27762600	0.02021800
C	-4.63962300	-0.22550400	-0.31473300
H	-4.75579000	1.89191700	-0.67182800
H	-4.18460500	-2.28485100	0.08615100
C	-1.53347700	-2.23848400	0.65457100
H	-0.62072900	-2.29689800	0.05388200
H	-1.21339600	-2.15587500	1.69870900
H	-2.07290100	-3.18053900	0.55025700
C	-2.20093200	2.71811200	-0.28453800
H	-1.77958900	3.06948300	0.66173100
H	-1.40250900	2.77323500	-1.03024800
H	-2.98416000	3.41657900	-0.58105900
C	-6.10939900	-0.46603300	-0.55826400
H	-6.70054600	-0.18215000	0.31890700
H	-6.47468100	0.12475200	-1.40189800
H	-6.31174200	-1.51823300	-0.76632600
H	0.29528800	-0.08522300	-1.49397900

[Im^{Mes,iBu}]

O	-3.39690900	2.34133900	-0.16086600
N	-1.22163000	-0.40307500	0.23818300
N	0.94659500	-0.45216800	0.48883000
C	-0.05311100	-0.20299800	-0.37297400
C	0.38537100	-0.79528700	1.71131000
H	0.99268600	-1.02792600	2.56813900
C	-0.96444900	-0.75927500	1.55231300
H	-1.75403900	-0.94541100	2.25705800
C	-2.48768700	0.11425400	-0.32333500
H	-2.50595900	-0.22017300	-1.36312300
C	-2.34995800	1.72900200	-0.38276600

O	-1.19815600	2.11589900	-0.68035100
C	-3.70870500	-0.41457400	0.42759700
H	-3.83836900	0.17606000	1.33905700
C	-5.01912500	-0.34549800	-0.38540600
H	-5.08171500	0.66382500	-0.80024400
C	2.35051400	-0.26518400	0.19991800
C	3.12595700	-1.38022200	-0.14368400
C	2.87201700	1.03614100	0.26120500
C	4.47559200	-1.16480000	-0.42731300
C	4.22763700	1.19185200	-0.03933800
C	5.04341200	0.11076600	-0.37960300
H	5.09379500	-2.01371200	-0.70116600
H	4.65320100	2.18961800	-0.00667000
C	2.02097100	2.22156400	0.64676800
H	1.10333400	2.30204000	0.05659200
H	1.71229200	2.15445400	1.69535300
H	2.58704500	3.14624800	0.52872700
C	2.53180000	-2.76568600	-0.22472900
H	2.12916300	-3.09338400	0.73791400
H	1.71096200	-2.80994600	-0.94652000
H	3.28780700	-3.48841700	-0.53359900
C	6.51105500	0.31392500	-0.66619700
H	7.09566100	0.27374200	0.25924000
H	6.89871200	-0.45903100	-1.33306600
H	6.69546400	1.28674700	-1.12696500
H	0.07083000	0.13054800	-1.38585400
H	-3.54456200	-1.46101800	0.72224300
C	-6.22024100	-0.55143600	0.54658600
H	-7.16134900	-0.49474500	-0.00803200
H	-6.18171200	-1.53352200	1.03351300
H	-6.24667000	0.21326400	1.32755900
C	-5.04765000	-1.36626400	-1.53194200
H	-4.22368200	-1.22823000	-2.23832300
H	-4.99145900	-2.39210500	-1.14842100
H	-5.97608800	-1.27781600	-2.10324100

*Compounds **1’** (carbene isomer)*

R = H

O	3.89937300	-1.44097600	0.15084700
N	2.17054100	0.66782800	0.00618700
N	0.09008900	0.37078400	0.33011800
C	1.01725900	0.38411200	-0.67458500
C	0.64726700	0.63828500	1.58271300
H	0.06248700	0.67024000	2.48669600
C	1.96996500	0.82717700	1.37617900
H	2.76377700	1.04783700	2.07032800
C	3.44914600	0.78316000	-0.65831400
H	3.25213200	0.85524700	-1.72949700
C	4.42909100	-0.36645500	-0.46468200
O	5.57124200	-0.31431300	-0.84510000
C	-1.31068000	0.11529200	0.11685800
C	-2.17460500	1.19522600	-0.10953100
C	-1.77146000	-1.20750300	0.13261300
C	-3.53021700	0.92240000	-0.30550900
C	-3.13604100	-1.43014600	-0.06902800
C	-4.03108400	-0.38090300	-0.28424300
H	-4.20854300	1.75036500	-0.48847600
H	-3.50448000	-2.45147800	-0.06413000
C	-0.82291700	-2.36291300	0.33487400
H	-0.06282700	-2.38360500	-0.45051800
H	-0.29189300	-2.28809700	1.28821000
H	-1.36282900	-3.31112600	0.32052900
C	-1.65678900	2.61101700	-0.16986100
H	-1.18925000	2.91268600	0.77204300
H	-0.89810400	2.71455700	-0.95023300
H	-2.46843200	3.30870100	-0.38237000
C	-5.50610400	-0.64508500	-0.47013100
H	-6.04083700	-0.57242100	0.48339200
H	-5.95750600	0.07873600	-1.15292700
H	-5.68440700	-1.64578900	-0.87000100
H	4.59694600	-2.11275500	0.19925800

H	3.96744500	1.69695000	-0.35653400

R = Me

O	3.59426300	-1.86598800	-0.30833500
N	1.95678900	0.34448900	-0.00320800
N	-0.13658800	0.17919700	0.33672400
C	0.77425300	0.23938200	-0.68261900
C	0.45990100	0.24395500	1.59691500
H	-0.10518300	0.20444600	2.51315500
C	1.79064700	0.35325400	1.38120300
H	2.60730000	0.42693800	2.08012800
C	3.23742300	0.47184300	-0.69051100
H	2.99268400	0.32204800	-1.74652700
C	4.20021500	-0.66022600	-0.31610700
O	5.37534500	-0.51602300	-0.09023100
C	3.87855000	1.84548300	-0.49830300
H	4.12419800	2.02996700	0.54954400
C	-1.55559900	0.05438600	0.12915100
C	-2.33145900	1.21411800	0.00116000
C	-2.12233500	-1.22470500	0.05162000
C	-3.70695500	1.06700800	-0.19227300
C	-3.50236400	-1.32090200	-0.14314500
C	-4.31159600	-0.18956600	-0.26177700
H	-4.31770300	1.95837300	-0.29913200
H	-3.95246900	-2.30663800	-0.21204100
C	-1.26866900	-2.46475400	0.14900200
H	-0.49433000	-2.46639100	-0.62249600
H	-0.75641500	-2.52837700	1.11361500
H	-1.87867300	-3.36188500	0.03078500
C	-1.70077200	2.58398100	0.04151100
H	-1.16359300	2.75509400	0.97882100
H	-0.97482300	2.70056400	-0.76775200
H	-2.46010400	3.36104500	-0.06076700
C	-5.80520400	-0.32092300	-0.44077300
H	-6.31738700	-0.32472400	0.52780600
H	-6.21174600	0.51034300	-1.02159100
H	-6.06503400	-1.25098200	-0.95150400
H	4.27243100	-2.52527200	-0.09642100
H	4.80196500	1.91613800	-1.07368200
H	3.18439300	2.61559300	-0.83835800

R = ^{i}Pr

O	3.76759100	-1.66591700	0.96091500
N	1.50796400	-0.03411600	0.16384400
N	-0.60762800	-0.02117600	0.41044400
C	0.34812700	-0.00612900	-0.56732500
C	-0.06343800	-0.05584700	1.69564800
H	-0.66937500	-0.07775900	2.58607400
C	1.27991300	-0.06308800	1.54039900
H	2.06768600	-0.11067200	2.27115900
C	2.81810700	-0.00515600	-0.49050800
H	2.59936300	0.09297900	-1.55358900
C	3.53308600	-1.34811200	-0.33812500
O	3.87948900	-2.05292200	-1.24921400
C	3.70500700	1.18067100	-0.02990000
H	3.86227800	1.07949300	1.04997900
C	5.07746200	1.13666200	-0.71780400
H	5.68320300	1.98880600	-0.39978500
H	5.63601400	0.22811100	-0.47872200
H	4.97381400	1.18765300	-1.80613900
C	3.00215300	2.51686900	-0.30028700
H	2.83521800	2.65997300	-1.37237100
H	2.03438400	2.57695800	0.20004200
H	3.62062800	3.34463400	0.05705800
C	-2.02186000	-0.01305400	0.14188700
C	-2.69756300	1.21231100	0.07385400
C	-2.68569900	-1.23240900	-0.04856400
C	-4.07232800	1.19279900	-0.17406700
C	-4.06042000	-1.20099400	-0.29449800

C	-4.77223500	-0.00141500	-0.35523800
H	-4.60544300	2.13682500	-0.23454900
H	-4.58446800	-2.13937500	-0.44895100
C	-1.93801300	-2.54226200	-0.01569900
H	-1.16529400	-2.56802600	-0.78864900
H	-1.43508900	-2.69657500	0.94343100
H	-2.61938300	-3.37873200	-0.17945000
C	-1.96270600	2.51999700	0.23502300
H	-1.47576300	2.59358200	1.21186100
H	-1.17959300	2.62086700	-0.52126500
H	-2.64926200	3.36232600	0.13548500
C	-6.26368400	0.00239000	-0.59069400
H	-6.81020700	-0.06616800	0.35644200
H	-6.58622500	0.91969300	-1.08861400
H	-6.57166300	-0.84507500	-1.20755600
H	4.20472900	-2.53117100	0.96987300

R = ^{i}Bu

O	-3.20071100	2.10087400	1.00303300
N	-1.09274500	0.25300000	0.29257800
N	1.02460100	0.10184600	0.46522900
C	0.02932700	0.06089700	-0.47096300
C	0.53962300	0.30810500	1.75810900
H	1.18280200	0.37372100	2.61980700
C	-0.80507000	0.40183900	1.65026100
H	-1.55454400	0.58350800	2.39976200
C	-2.42528100	0.24733200	-0.31556300
H	-2.24440600	0.04769500	-1.36927700
C	-3.08516200	1.62489300	-0.26214600
O	-3.49774700	2.22536200	-1.21998600
C	-3.33164100	-0.84183900	0.29330200
H	-3.43830300	-0.65415700	1.36607600
C	2.42134900	-0.04679100	0.15078700
C	2.99527100	-1.32468600	0.17584000
C	3.17011200	1.09137700	-0.17702900
C	4.35571600	-1.44053300	-0.11968600
C	4.52679100	0.92531300	-0.46495500
C	5.13905700	-0.32910800	-0.43575800
H	4.81047600	-2.42649400	-0.10888400
H	5.11605700	1.79929300	-0.72566000
C	2.52816800	2.45474100	-0.24686700
H	1.73862200	2.47288700	-1.00299100
H	2.06512500	2.73360100	0.70402000
H	3.26743100	3.21639800	-0.50038300
C	2.16724400	-2.54732200	0.48500400
H	1.70717300	-2.48534900	1.47547100
H	1.35459800	-2.65841800	-0.23801000
H	2.78327800	-3.44756700	0.45360400
C	6.61518400	-0.47682100	-0.71737400
H	7.20195600	-0.36703700	0.20139500
H	6.84475500	-1.45927100	-1.13631500
H	6.96493700	0.28244300	-1.42076500
H	-3.62133500	2.97240200	0.94457900
H	-2.79596000	-1.79194000	0.18756400
C	-4.73200700	-0.96948200	-0.33643700
H	-5.24129900	-0.00127600	-0.24399100
C	-5.55667000	-2.00091300	0.44727600
H	-6.57115700	-2.07661200	0.04632400
H	-5.10042000	-2.99488200	0.38573400
H	-5.63331200	-1.73320600	1.50530000
C	-4.67817100	-1.33377500	-1.82736700
H	-4.18914800	-0.56137200	-2.42479100
H	-4.13786200	-2.27462500	-1.97920700
H	-5.68783300	-1.46291500	-2.22699200

Compounds [Im$^{Ar',Me}$]

Ar' = p-$H_2NC_6H_4$

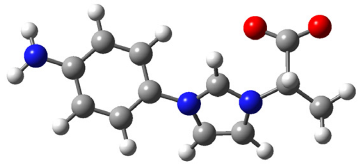

O	4.37442600	1.89583700	-0.09521800
N	1.95755500	-0.74875200	0.30082900
N	-0.20358200	-0.80072900	-0.00796700
C	0.80441100	-0.14073800	0.58821200
C	0.33839800	-1.85977800	-0.72636500
H	-0.27342200	-2.51742700	-1.31742800
C	1.68290900	-1.82119100	-0.53095500
H	2.45757900	-2.45712200	-0.91768000
C	3.26116000	-0.10281600	0.56723900
H	3.33355700	0.01862100	1.65148100
C	3.24700500	1.40003300	-0.03555800
O	2.11306400	1.85281200	-0.30316600
C	4.42912800	-0.93614000	0.06740700
H	4.39531100	-1.06589400	-1.01788000
C	-1.57507200	-0.38523100	0.00966500
C	-2.58634200	-1.30127600	0.29209200
C	-1.88964500	0.94602900	-0.26488700
C	-3.91176300	-0.88941300	0.29614700
C	-3.21386200	1.35902900	-0.24416400
C	-4.24885700	0.44932200	0.03179300
H	-4.69402400	-1.60512700	0.52450300
H	-3.45127400	2.39606500	-0.45492100
H	0.69381100	0.73179800	1.20231500
H	4.48376500	-1.91826000	0.54874100
H	5.33765800	-0.37993300	0.29122700
H	-2.33968800	-2.33125900	0.52279800
H	-1.09957400	1.64811200	-0.50814300
N	-5.56725300	0.87322700	0.09555600
H	-5.79191100	1.74687100	-0.35479900
H	-6.28357500	0.17116300	-0.00582000

Ar' = p-HOC_6H_4

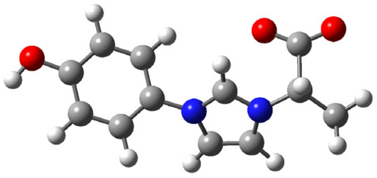

O	4.35080900	1.89363800	-0.11234500
N	1.94897000	-0.75589800	0.31135100
N	-0.21098800	-0.79774700	-0.00450900
C	0.79821700	-0.14336100	0.59719100
C	0.32896600	-1.85549700	-0.72762400
H	-0.28318100	-2.50725100	-1.32488200
C	1.67257100	-1.82237000	-0.52817300
H	2.44619700	-2.45817800	-0.91726500
C	3.25158300	-0.10629700	0.57169100
H	3.32613500	0.02211200	1.65494200
C	3.22683900	1.39320300	-0.04097200
O	2.08802800	1.83814600	-0.30318300
C	4.42112200	-0.93765800	0.07291400
H	4.38539200	-1.07254800	-1.01164400
C	-1.58030200	-0.37656800	0.01042600
C	-2.59147400	-1.29263300	0.28471400

C	-1.88475900	0.95950300	-0.25982800
C	-3.91834900	-0.87381000	0.28423600
C	-3.20706600	1.37975500	-0.24275900
C	-4.22808100	0.46391600	0.02541500
H	-4.70872000	-1.58583500	0.49913200
H	-3.46476700	2.41062100	-0.45031800
H	0.68805600	0.72377800	1.21896800
H	4.48056000	-1.91707000	0.55900700
H	5.32828700	-0.37717900	0.29168600
H	-2.34833200	-2.32383600	0.51205700
H	-1.08844800	1.65686600	-0.49592600
O	-5.50571600	0.93669700	0.01818900
H	-6.12762000	0.22709700	0.21168100

Ar’ = *p*-MeC_6H_4

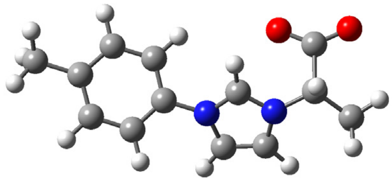

O	4.37959900	1.90201600	-0.12700800
N	1.96918500	-0.74029300	0.31003100
N	-0.19081800	-0.79875200	-0.00102100
C	0.81700400	-0.12460100	0.58174000
C	0.35152700	-1.87438800	-0.69504700
H	-0.25871200	-2.54440000	-1.27365600
C	1.69535400	-1.83027300	-0.49974100
H	2.47062100	-2.47309100	-0.87368800
C	3.27244200	-0.08978900	0.56639500
H	3.34611700	0.04672000	1.64871500
C	3.25371000	1.40562800	-0.05619000
O	2.11740100	1.85283700	-0.32438400
C	4.44089400	-0.92740100	0.07538900
H	4.40350800	-1.07434200	-1.00757800
C	-1.56381600	-0.38946400	0.01299600
C	-2.56810400	-1.32190600	0.25667400
C	-1.87599600	0.94918200	-0.22217000
C	-3.89702400	-0.90646400	0.25824500
C	-3.20819700	1.34749900	-0.19924200
C	-4.24080600	0.43075900	0.03367200
H	-4.67717300	-1.63448900	0.45228100
H	-3.44731700	2.38995700	-0.38004700
C	-5.68283900	0.87353000	0.01887300
H	-6.04020200	1.00151700	-1.00830300
H	-6.33007300	0.14126500	0.50510400
H	-5.80932400	1.83153800	0.52878000
H	0.70565400	0.75743300	1.18176800
H	4.50088600	-1.90138400	0.57222500
H	5.34833000	-0.36464100	0.28704000
H	-2.31605300	-2.35569400	0.46250700
H	-1.08571200	1.66022800	-0.43664100

Ar’ = C_6H_5

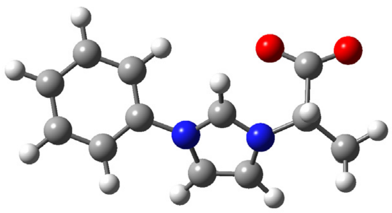

O	4.05254900	1.73888100	-0.13493300

N	1.51435300	-0.77614700	0.31946300
N	-0.64573500	-0.70246500	0.01206700
C	0.40223100	-0.09277000	0.59555200
C	-0.17037100	-1.80590400	-0.68871600
H	-0.82077300	-2.43490500	-1.26962600
C	1.17371700	-1.84334900	-0.49602000
H	1.90845600	-2.52945200	-0.87492500
C	2.85277000	-0.19643700	0.56373700
H	2.94480200	-0.06483400	1.64520300
C	2.90447200	1.29777100	-0.06004800
O	1.79013100	1.79922500	-0.32643800
C	3.97074000	-1.09260400	0.05903000
H	3.91599100	-1.23217600	-1.02414900
C	-1.98895200	-0.20435300	0.02300300
C	-3.05079300	-1.07788200	0.25066200
C	-2.20460900	1.15460700	-0.20520300
C	-4.35107600	-0.57944100	0.24504900
C	-3.50935000	1.64176100	-0.19089200
C	-4.58164100	0.77865200	0.02931200
H	-5.18220500	-1.25177400	0.42220000
H	-3.68517300	2.69614000	-0.36779700
H	0.34331200	0.78851300	1.20396200
H	3.98322900	-2.07064500	0.55126000
H	4.90866200	-0.57992200	0.26499300
H	-2.86165000	-2.12687100	0.44582800
H	-1.36427900	1.80853400	-0.41101900
H	-5.59496100	1.16253200	0.03153600

Ar’ = *p*-FC_6H_4

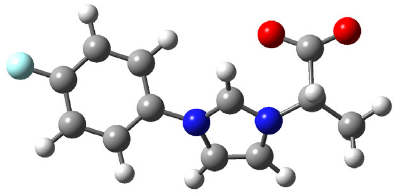

O	4.29024600	1.91726500	-0.15599200
N	1.94704600	-0.76697000	0.33084000
N	-0.21247300	-0.81275500	0.01283800
C	0.79475200	-0.16046900	0.62129800
C	0.33174200	-1.86155900	-0.72226900
H	-0.27723900	-2.50965600	-1.32672500
C	1.67448100	-1.82499500	-0.52170800
H	2.45070700	-2.45319500	-0.91809500
C	3.24107300	-0.09514400	0.57378300
H	3.32214400	0.04806100	1.65469600
C	3.17874400	1.39601800	-0.05800000
O	2.02604700	1.81545600	-0.30650100
C	4.42096800	-0.91072000	0.07417200
H	4.37931600	-1.05769800	-1.00852600
C	-1.57858100	-0.38479700	0.02027300
C	-2.59670200	-1.30615400	0.25988000
C	-1.86427000	0.95947700	-0.22265200
C	-3.92190400	-0.88212900	0.25278900
C	-3.18757700	1.38972100	-0.21221600
C	-4.18959800	0.45950100	0.02118700
H	-4.73666400	-1.57046600	0.43681200
H	-3.44345600	2.42490300	-0.39793900
H	0.67930000	0.69439000	1.25874000
H	4.50115000	-1.88338800	0.57039500
H	5.32022200	-0.33258200	0.27942600
H	-2.35838800	-2.34273500	0.46563200
H	-1.05864800	1.65386600	-0.43469000
F	-5.47439800	0.87247900	0.02169500

Ar’ = *p*-ClC_6H_4

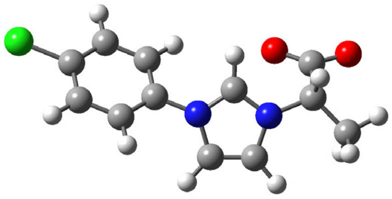

O	-4.61685400	-2.02548700	-0.17005400
N	-2.36641200	0.73364100	0.33346600
N	-0.21151300	0.86639700	0.00923000
C	-1.19130700	0.16651200	0.61081600
C	-0.79790400	1.90576100	-0.70695800
H	-0.21677200	2.58521900	-1.30426400
C	-2.13749900	1.81393100	-0.50387500
H	-2.93865000	2.41720900	-0.88927000
C	-3.63428800	0.01412600	0.57704100
H	-3.70338600	-0.14261000	1.65691600
C	-3.52339300	-1.46811000	-0.06901100
O	-2.35760000	-1.84598100	-0.32284300
C	-4.84535200	0.79251700	0.09296500
H	-4.81442300	0.95450200	-0.98795200
C	1.17069300	0.49815900	0.01464500
C	2.14962200	1.46541000	0.23273400
C	1.51571700	-0.83500700	-0.20678300
C	3.49139400	1.09829900	0.22697500
C	2.85698900	-1.20506500	-0.19532400
C	3.83339500	-0.23548600	0.01762100
H	4.26335900	1.83727700	0.39718400
H	3.14029400	-2.23531400	-0.36670400
H	-1.04126100	-0.69076200	1.23763900
H	-4.95871400	1.75524600	0.60193500
H	-5.72188100	0.17930800	0.29482700
H	1.86997300	2.49467600	0.42313100
H	0.74319700	-1.56953500	-0.40657200
Cl	5.52299800	-0.69882500	0.02014000

Compounds [Im$^{Ar’,Me}$] (carbene isomer)

Ar’ = *p*-$H_2NC_6H_4$

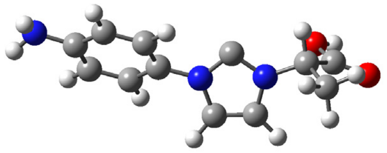

O	3.41585900	-1.16424700	-1.53353600
N	1.76944500	0.22845900	0.22397400
N	-0.32560000	-0.12195000	0.38006800
C	0.58438800	0.62132100	-0.32763200
C	0.28253400	-0.94021500	1.33400400
H	-0.26880300	-1.57835500	2.00262100
C	1.61224600	-0.71716200	1.23607800
H	2.43252200	-1.14012100	1.79201300
C	3.04774400	0.78963700	-0.20177600
H	2.80048000	1.38780900	-1.08370100
C	4.00981200	-0.30183700	-0.68349500
O	5.17355000	-0.37699200	-0.37844100
C	3.68667400	1.67619400	0.86584600
H	3.93351400	1.10638000	1.76393000
C	-1.73555600	-0.05596300	0.15959500
C	-2.32913400	1.14980800	-0.21556200
C	-2.53485600	-1.18836400	0.31000000
C	-3.69864700	1.22008600	-0.43084000
C	-3.90927400	-1.11517800	0.10754000

C	-4.51556400	0.09128500	-0.26581300
H	-4.14344500	2.16433200	-0.72829500
H	-4.51406500	-2.00904400	0.22043800
H	4.08920100	-1.80335700	-1.81191800
H	4.60935900	2.11851500	0.48874000
H	2.99071100	2.47277900	1.13359400
H	-1.70399600	2.02353000	-0.34468100
H	-2.08691100	-2.14131000	0.56601000
N	-5.88699900	0.15443500	-0.53028700
H	-6.45288900	-0.54838100	-0.07807400
H	-6.30107400	1.07284700	-0.46881400

Ar’ = *p*-HOC_6H_4

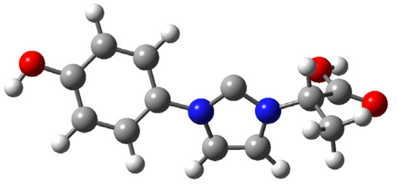

O	3.39828500	1.58916000	-1.04336500
N	1.75706200	-0.25843800	0.20658700
N	-0.33822800	-0.37547300	-0.15353700
C	0.57667200	0.27092300	0.63892400
C	0.26344200	-1.26997500	-1.04129600
H	-0.28736800	-1.84189200	-1.76782600
C	1.59310300	-1.19931300	-0.80918900
H	2.41125400	-1.71433500	-1.28470400
C	3.03718400	0.10925600	0.80388100
H	2.79212000	0.93110800	1.48318300
C	4.00099500	0.69242200	-0.23568700
O	5.17304200	0.42265300	-0.31295500
C	3.67434700	-1.03791000	1.58613600
H	3.91275500	-1.88166800	0.93538900
C	-1.74521200	-0.15221200	-0.06482400
C	-2.64426600	-1.19459300	-0.27818700
C	-2.22946800	1.12142100	0.24490000
C	-4.01725200	-0.96591500	-0.20086700
C	-3.59529000	1.34840300	0.33687500
C	-4.49591300	0.30607000	0.10842400
H	-4.70966100	-1.78572300	-0.36669300
H	-3.97968600	2.33210500	0.57716400
H	4.07457300	1.93221000	-1.64706100
H	4.60150400	-0.70722300	2.05487800
H	2.98141700	-1.37389200	2.35914600
H	-2.28302300	-2.19489500	-0.48440900
H	-1.52211800	1.92111400	0.42017200
O	-5.83175800	0.59415900	0.20406100
H	-6.35019600	-0.19877100	0.03293500

Ar’ = *p*-MeC_6H_4

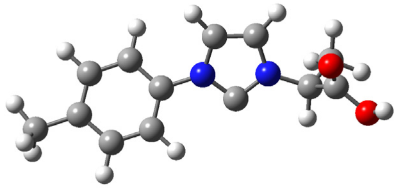

O	-4.88520600	-1.50221700	-0.20718900
N	-1.74480900	0.21645900	0.22193100
N	0.35891300	0.42615300	-0.04371400
C	-0.57406900	-0.44315200	0.46049700
C	-0.22389800	1.58233600	-0.57139000
H	0.34019700	2.36315100	-1.05072400

C	-1.55739400	1.45048700	-0.40105400
H	-2.36647900	2.08828700	-0.70956100
C	-3.01753500	-0.33627900	0.66380200
H	-2.77469200	-1.31568100	1.07609600
C	-3.97861300	-0.54121700	-0.50362100
O	-3.98808600	0.07110400	-1.53952500
C	-3.68726200	0.52139300	1.75232000
H	-3.93823200	1.51573300	1.37582800
C	1.76147400	0.16596800	-0.02227800
C	2.67858000	1.21231100	0.05368400
C	2.22459400	-1.15088800	-0.07530700
C	4.04675200	0.94133900	0.05984500
C	3.58985700	-1.40481600	-0.05531200
C	4.52949000	-0.36702000	0.00574000
H	4.74658800	1.76838600	0.12098300
H	3.93306000	-2.43396700	-0.09595600
C	6.00992500	-0.66232400	0.00714400
H	6.32209400	-1.12630100	-0.93411900
H	6.59681700	0.24897500	0.13882100
H	6.27708900	-1.35290100	0.81267300
H	-5.49642000	-1.56511300	-0.95709700
H	-4.60220300	0.04098200	2.10303600
H	-3.00138600	0.63012700	2.59404600
H	2.33527100	2.23700800	0.13041100
H	1.50450100	-1.95689800	-0.12265800

Ar’ = C_6H_5

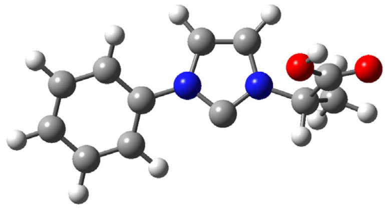

O	2.92786800	-1.20814900	-1.42892800
N	1.33953300	0.21524600	0.27077700
N	-0.76420900	-0.11090700	0.34697600
C	0.18025000	0.64896400	-0.29890900
C	-0.19704300	-0.97880000	1.28394500
H	-0.77509900	-1.63548500	1.91008200
C	1.13651600	-0.76941000	1.23724400
H	1.93203100	-1.22741100	1.80087100
C	2.63765000	0.76812400	-0.09906600
H	2.41405400	1.45457400	-0.92185200
C	3.57884100	-0.28662000	-0.69071700
O	4.77680500	-0.27767900	-0.55806800
C	3.30034400	1.53744900	1.04430500
H	3.51459800	0.88549400	1.89399700
C	-2.16177600	-0.01596900	0.07580800
C	-2.70032900	1.19798000	-0.35639900
C	-2.98959000	-1.12867200	0.23633100
C	-4.06279800	1.29204500	-0.62090000
C	-4.35535400	-1.02038900	-0.01957000
C	-4.89812000	0.18801600	-0.44901200
H	-4.47455400	2.23707800	-0.95687300
H	-4.99092200	-1.88970100	0.10563200
H	3.59793800	-1.80215700	-1.80021100
H	4.24307900	1.97004100	0.70985800
H	2.63465000	2.33614700	1.37504700
H	-2.04053300	2.04516700	-0.48677900
H	-2.57170400	-2.08226300	0.53526900
H	-5.95975200	0.26846900	-0.65170700

Ar’ = p-FC_6H_4

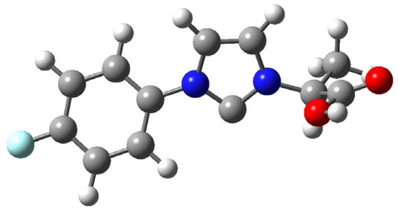

O	-3.38506200	-1.50449500	-1.13679900
N	-1.74613100	0.25131200	0.20982900
N	0.34866700	0.38159500	-0.14843700
C	-0.56690500	-0.29402200	0.62041600
C	-0.25386500	1.30956100	-1.00185500
H	0.29423900	1.90841700	-1.70823900
C	-1.58257300	1.22898700	-0.77099200
H	-2.40095600	1.76039500	-1.22756200
C	-3.02558800	-0.13609500	0.79658500
H	-2.77967400	-0.98091300	1.44712100
C	-3.99542300	-0.68584100	-0.25529000
O	-5.17813900	-0.45709700	-0.28078900
C	-3.65902900	0.98331200	1.62145300
H	-3.89801100	1.85042800	1.00219800
C	1.75359000	0.15453000	-0.06383100
C	2.65386200	1.19855300	-0.28162800
C	2.22886000	-1.12171000	0.24678700
C	4.02645500	0.96983500	-0.20684800
C	3.59700600	-1.35364500	0.33740300
C	4.47204400	-0.30378300	0.10293600
H	4.74005600	1.76748600	-0.36990800
H	3.98569500	-2.33532100	0.57745500
H	-4.06722800	-1.83657300	-1.74013900
H	-4.58522000	0.63648700	2.07989600
H	-2.96383000	1.29063000	2.40422200
H	2.29248900	2.19844500	-0.48746700
H	1.51722600	-1.91679300	0.42366600
F	5.80694000	-0.53093200	0.18210200

Ar’ = p-ClC_6H_4

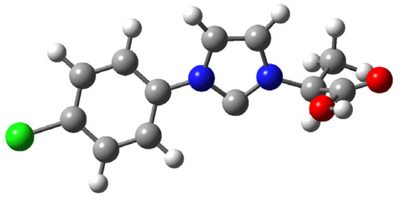

O	-3.76425700	-1.46363200	-1.21595300
N	-2.16197600	0.24044200	0.22151700
N	-0.07248000	0.43342400	-0.14151600
C	-0.96970600	-0.29949200	0.59758700
C	-0.70043000	1.39021800	-0.94393200
H	-0.17117900	2.03544100	-1.62315600
C	-2.02524100	1.26886500	-0.71054000
H	-2.85765100	1.80382400	-1.13645700
C	-3.42928500	-0.20310700	0.79472300
H	-3.16052900	-1.07005400	1.40609800
C	-4.39267800	-0.72986800	-0.27455800
O	-5.58461300	-0.55414300	-0.26502400
C	-4.08449900	0.86258000	1.67240100
H	-4.34987900	1.75014400	1.09402900
C	1.33573500	0.23154400	-0.07458500
C	2.21642500	1.29261700	-0.28858000
C	1.83891300	-1.03894300	0.21337400
C	3.59281300	1.08612300	-0.23218100
C	3.21150000	-1.24669600	0.28324200

C	4.08025600	-0.18323100	0.05419100
H	4.27680800	1.90883100	-0.39542900
H	3.60431800	-2.23040900	0.50597200
H	-4.44317100	-1.79003600	-1.82607800
H	-4.99826800	0.47205500	2.12038000
H	-3.39159300	1.15230300	2.46383000
H	1.83896400	2.29002000	-0.47708600
H	1.14532000	-1.85044200	0.38776200
Cl	5.81742400	-0.44883500	0.13065200

[NHC^{Mes,R}]$^-$ ligands

[NHC^{Mes,H}]$^-$

O	4.57518100	-0.67784600	0.94539500
N	2.16344200	0.35086300	-0.18991100
N	0.07442900	0.24071000	0.23173500
C	0.96701800	0.14548900	-0.81141500
C	0.70846300	0.49983600	1.45379300
H	0.17363000	0.58251200	2.38555200
C	2.03322900	0.56357600	1.17532000
H	2.89806800	0.65770000	1.80798000
C	3.44506000	0.39938700	-0.91150800
H	3.26810300	-0.04762500	-1.88740000
C	4.68797000	-0.29562200	-0.24057400
O	5.67100900	-0.33804900	-1.00985800
C	-1.33683100	0.07105500	0.07982300
C	-2.15920500	1.20014700	-0.04444000
C	-1.87462400	-1.22460100	0.04834400
C	-3.53744400	1.01061400	-0.18119800
C	-3.25768100	-1.36740900	-0.09278900
C	-4.10587200	-0.26388300	-0.20225700
H	-4.17816400	1.88198200	-0.28673200
H	-3.67920900	-2.36851900	-0.12289400
C	-0.97573500	-2.43137700	0.14088800
H	-0.28436200	-2.45950300	-0.70523400
H	-0.35743500	-2.40041200	1.04211400
H	-1.56275900	-3.35264500	0.15197800
C	-1.56448400	2.58628600	-0.06590700
H	-1.08506900	2.83445900	0.88540300
H	-0.79052800	2.65900900	-0.83428900
H	-2.33435700	3.33467700	-0.26766200
C	-5.60180200	-0.44457200	-0.32009600
H	-6.07466600	-0.49968000	0.66757000
H	-6.06261500	0.38952500	-0.85596100
H	-5.85120000	-1.36661800	-0.85205500
H	3.71756800	1.44773400	-1.07333600

[NHC^{Mes,Me}]$^-$

O	4.47663600	-0.66401700	1.12073800
N	1.95356400	0.15603200	-0.04915400
N	-0.14872500	0.09072700	0.31376900
C	0.76425700	0.09327600	-0.71508200
C	0.46532700	0.15572000	1.57114300
H	-0.08954700	0.14047700	2.49487000
C	1.79838400	0.19952700	1.33134500
H	2.66133500	0.18584000	1.97560100
C	3.25033200	0.24290000	-0.76044000
H	3.04833300	-0.15027900	-1.75454000
C	4.42373700	-0.60580000	-0.13157900
O	5.21062500	-1.06884700	-0.98314000
C	3.69003400	1.70871400	-0.87205200
H	3.87974600	2.12995600	0.11974000
C	-1.56212300	0.00714500	0.11522500
C	-2.32358100	1.18387600	0.07113600
C	-2.16280000	-1.25131400	-0.03851100
C	-3.70571100	1.07862600	-0.11002400
C	-3.54748400	-1.30973500	-0.21963600
C	-4.33641500	-0.15839900	-0.25158100

H	-4.29922200	1.98796700	-0.15176900
H	-4.01764700	-2.28135500	-0.34530800
C	-1.32794600	-2.50675200	-0.03279400
H	-0.59008000	-2.48031900	-0.83867400
H	-0.76466500	-2.60771100	0.89908800
H	-1.95693600	-3.39145100	-0.15555800
C	-1.65839700	2.53298100	0.18300800
H	-1.14072600	2.64799900	1.13944000
H	-0.90180200	2.65216600	-0.59703600
H	-2.39253900	3.33647900	0.08878900
C	-5.83613100	-0.25088800	-0.41472700
H	-6.33592600	-0.35509900	0.55555200
H	-6.24053800	0.64400900	-0.89515700
H	-6.11807400	-1.11614100	-1.02067300
H	2.92191300	2.31050600	-1.36975400
H	4.61552400	1.76328200	-1.45028900

[NHC^{Mes,iPr}]$^-$

O	3.97162400	-1.34215800	1.26384500
N	1.52847000	-0.13797100	0.13910000
N	-0.58804200	-0.09303500	0.41154300
C	0.36965100	-0.03527200	-0.57573600
C	-0.03092100	-0.22749600	1.68930000
H	-0.62773300	-0.31487200	2.58233100
C	1.31127400	-0.25568800	1.50752800
H	2.13746700	-0.41341500	2.18075400
C	2.87026800	-0.11569600	-0.48288900
H	2.68815800	-0.24324900	-1.54972900
C	3.76267200	-1.31875700	0.02442900
O	4.18833400	-2.07921000	-0.86746500
C	3.58285000	1.23444700	-0.22871000
H	3.72582400	1.31276200	0.85561700
C	4.97156100	1.23703300	-0.88490700
H	5.48833900	2.18255800	-0.68406100
H	5.58971700	0.41862300	-0.51252500
H	4.88884900	1.12261900	-1.97164100
C	2.76253200	2.43994500	-0.70949700
H	2.59571800	2.39364900	-1.79142100
H	1.78216400	2.48268700	-0.23037500
H	3.29008400	3.37554600	-0.48916500
C	-1.99345700	-0.05387100	0.14983800
C	-2.68621600	1.15891000	0.27308500
C	-2.65402600	-1.23110700	-0.23364800
C	-4.06228900	1.16984500	0.02658000
C	-4.02955300	-1.17311700	-0.47363900
C	-4.75218000	0.01421100	-0.34228400
H	-4.60274000	2.10858800	0.11388900
H	-4.54584700	-2.08030500	-0.77572600
C	-1.89108200	-2.52021400	-0.40314500
H	-1.12385100	-2.41398300	-1.17423100
H	-1.36805400	-2.79829400	0.51605000
H	-2.56381000	-3.33525600	-0.67972300
C	-1.95566100	2.43070900	0.62571100
H	-1.46799200	2.35972600	1.60172200
H	-1.16685500	2.63342200	-0.10370200
H	-2.64296800	3.27964600	0.64461500
C	-6.24555000	0.04183800	-0.57335200
H	-6.79378900	-0.21615900	0.34039400
H	-6.58166500	1.03379000	-0.88656000
H	-6.54291800	-0.67443100	-1.34404800

[NHC^{Mes,iBu}]$^-$

O	3.45425800	-1.75873500	1.48149300
N	1.10647700	-0.43239500	0.30113900
N	-1.01159900	-0.23426200	0.46962000
C	0.00805500	-0.12613900	-0.44776800
C	-0.54791800	-0.59246900	1.74183700
H	-1.20456100	-0.74766500	2.58207400
C	0.79667600	-0.71370700	1.62711700

H	1.57030400	-1.02008700	2.31189800
C	2.47740600	-0.41139500	-0.25954700
H	2.33352400	-0.51408900	-1.33236200
C	3.38357300	-1.60280700	0.23728100
O	3.97893900	-2.21479000	-0.67422900
C	3.13989800	0.94085300	0.06365900
H	3.17636200	1.04317800	1.15402700
C	-2.38891700	-0.02755100	0.14576300
C	-2.98929100	1.20572200	0.43727100
C	-3.11507000	-1.06112500	-0.46550400
C	-4.34070300	1.38101100	0.12501500
C	-4.46230700	-0.84048200	-0.76386500
C	-5.09495300	0.36917200	-0.47063000
H	-4.80836600	2.33752800	0.34226100
H	-5.02813600	-1.63562000	-1.24186600
C	-2.44993000	-2.36961500	-0.80947400
H	-1.63232600	-2.21017600	-1.51691100
H	-2.00637300	-2.83557100	0.07487300
H	-3.16877900	-3.06678900	-1.24602500
C	-2.18470000	2.32985600	1.04137900
H	-1.82339200	2.07606100	2.04200800
H	-1.30016200	2.53820500	0.43389100
H	-2.78491400	3.23969200	1.11395600
C	-6.56232700	0.56889200	-0.77209400
H	-7.18935100	0.21371600	0.05415100
H	-6.79650200	1.62511500	-0.92885200
H	-6.86331400	0.01968800	-1.66831700
H	2.49358600	1.74351000	-0.31951500
C	4.56255000	1.12312600	-0.49582300
H	5.18423800	0.31787200	-0.08921600
C	5.14732700	2.46176600	-0.02032200
H	6.17917800	2.58863800	-0.36660200
H	4.56184700	3.30622600	-0.40513600
H	5.14917700	2.52858000	1.07257800
C	4.61100500	1.01882100	-2.02679300
H	4.32388200	0.01885800	-2.35673700
H	3.93793700	1.75004500	-2.49214100
H	5.62419200	1.21431400	-2.39645600

Model compounds {Ag[NHC^{Mes,R}]}$_2$

{Ag[NHC^{Mes,H}]}$_2$, **2a$_c$**

Ag	-2.12210700	-0.85554600	-0.50147300
O	-0.41658900	4.11613100	0.15216800
N	-2.25583300	2.24568900	-0.85495400
N	-4.12935500	1.50333900	-0.09648800
C	-2.89586000	1.10100100	-0.50615500
C	-4.25164300	2.88714800	-0.18773000
H	-5.15640400	3.39971200	0.09861200
C	-3.06628600	3.35260600	-0.65986600
H	-2.70046500	4.35165700	-0.82449900
C	-0.87858400	2.30626700	-1.34324600
H	-0.53435900	1.27770700	-1.45019500
C	0.06338700	3.14611700	-0.42757600
O	1.29919000	2.78257100	-0.43576700
C	-5.16308200	0.61992600	0.38252900
C	-6.08307000	0.08974300	-0.53621200
C	-5.22571300	0.33232300	1.75636100
C	-7.08333500	-0.75417500	-0.04319200
C	-6.24549800	-0.51657800	2.19656400
C	-7.17868400	-1.07345600	1.31520000
H	-7.80499400	-1.17286600	-0.74101900
H	-6.30911000	-0.74955400	3.25710000
Ag	2.12204000	0.85516800	-0.50158600
O	0.41672900	-4.11490300	0.15388100
N	2.25614200	-2.24610800	-0.85533800
N	4.12946200	-1.50341800	-0.09671400
C	2.89602500	-1.10128600	-0.50677400
C	4.25185900	-2.88724600	-0.18751800
H	5.15658700	-3.39966800	0.09918000
C	3.06660500	-3.35291500	-0.65970700
H	2.70090500	-4.35205100	-0.82410400
C	0.87899900	-2.30695100	-1.34384700

H	0.53479800	-1.27846900	-1.45161500
C	-0.06323100	-3.14601300	-0.42771200
O	-1.29920300	-2.78300700	-0.43764200
C	5.16306300	-0.61978300	0.38217000
C	6.08333100	-0.09010800	-0.53658300
C	5.22527800	-0.33143100	1.75586400
C	7.08346600	0.75405900	-0.04372500
C	6.24495600	0.51767800	2.19591000
C	7.17842000	1.07406100	1.31452400
H	7.80533500	1.17236700	-0.74156300
H	6.30825400	0.75123000	3.25633800
H	0.87695900	-2.76753100	-2.33927500
H	-0.87639500	2.76611900	-2.33901500
C	-5.99837600	0.40965700	-2.01013200
H	-6.03314300	1.48958800	-2.19554300
H	-5.06078900	0.04260400	-2.44445700
H	-6.82641100	-0.05389000	-2.55376200
C	-4.22497400	0.90807800	2.73048000
H	-3.21203500	0.54213100	2.52463300
H	-4.18295100	2.00186400	2.67628100
H	-4.48121500	0.62867800	3.75622900
C	-8.24789900	-2.01529900	1.81765100
H	-7.86843000	-3.04377500	1.87746100
H	-8.58854800	-1.73665100	2.82063400
H	-9.11795500	-2.02625600	1.15313300
C	5.99904100	-0.41082600	-2.01035100
H	6.03470600	-1.49082500	-2.19520800
H	5.06119500	-0.04475900	-2.44494300
H	6.82676300	0.05309900	-2.55413200
C	4.22422500	-0.90660000	2.73000800
H	3.21148100	-0.54012500	2.52412100
H	4.18162200	-2.00036500	2.67588900
H	4.48058500	-0.62724200	3.75573900
C	8.24751000	2.01613500	1.81681100
H	7.86782800	3.04452700	1.87669000
H	8.58836800	1.73755800	2.81974500
H	9.11746500	2.02727300	1.15216700

{Ag[NHC^{Mes,Me}]}$_2$, **2b$_c$**

Ag	-1.96964900	-0.86181200	-0.72430800
O	-0.25995400	4.46217500	-0.90017600
N	-2.15719300	2.26051300	-0.90286800
N	-3.78001700	1.46737500	0.27179000
C	-2.68889000	1.09590600	-0.45398300
C	-3.92104200	2.85179100	0.27334000
H	-4.72221900	3.34260900	0.80329300
C	-2.89726500	3.35051400	-0.46675900
H	-2.57750600	4.35621200	-0.68361600
C	-0.96094200	2.35092900	-1.76475700
H	-0.57468900	1.33165700	-1.81808800
C	0.09949400	3.30645100	-1.12920600
O	1.28022800	2.82821000	-0.94937600
C	-1.33557200	2.83862000	-3.16789500
H	-1.69602900	3.87031100	-3.12848700
C	-4.66430000	0.55485300	0.95278400
C	-5.78255100	0.05364200	0.26629200
C	-4.38368300	0.20263300	2.28304100
C	-6.62808000	-0.82416300	0.95124400
C	-5.26166700	-0.67857200	2.92219000
C	-6.38736200	-1.20089900	2.27698800
H	-7.49559400	-1.22553800	0.43215000
H	-5.05579300	-0.96636900	3.95068000
C	-3.16826300	0.73888000	3.00207500
H	-2.24127400	0.39577500	2.52731600
H	-3.14020500	1.83455900	2.99689800
H	-3.15782800	0.40427800	4.04318100
C	-6.06345500	0.43298200	-1.16880400
H	-6.13550400	1.51931400	-1.29717600
H	-5.26649500	0.08561700	-1.83702800
H	-7.00394700	-0.01066800	-1.50729600
C	-7.33534700	-2.12774800	3.00158400
H	-8.15133700	-1.56612700	3.47583700
H	-7.79247100	-2.84872600	2.31584200
H	-6.82278600	-2.68759100	3.79049000

Ag	1.96955700	0.86195400	-0.72442400
O	0.25983500	-4.46199500	-0.90110900
N	2.15707900	-2.26033400	-0.90363600
N	3.78006600	-1.46742700	0.27095400
C	2.68883300	-1.09581600	-0.45458900
C	3.92109700	-2.85184200	0.27220200
H	4.72234800	-3.34276500	0.80194600
C	2.89723500	-3.35041800	-0.46787700
H	2.57746100	-4.35607300	-0.68491100
C	0.96069900	-2.35057700	-1.76536500
H	0.57444500	-1.33129200	-1.81844100
C	-0.09964400	-3.30622300	-1.12984600
O	-1.28035000	-2.82801700	-0.94973400
C	1.33512200	-2.83799400	-3.16865400
H	1.69558300	-3.86969300	-3.12950000
C	4.66443100	-0.55504000	0.95202200
C	5.78260100	-0.05369700	0.26550200
C	4.38396800	-0.20308000	2.28238300
C	6.62821200	0.82397300	0.95053100
C	5.26202600	0.67799500	2.92160400
C	6.38764900	1.20044800	2.27637400
H	7.49566700	1.22544800	0.43141600
H	5.05626900	0.96559100	3.95017400
C	3.16863000	-0.73947200	3.00145000
H	2.24158600	-0.39632000	2.52683100
H	3.14060300	-1.83515100	2.99610600
H	3.15828500	-0.40503100	4.04260900
C	6.06334700	-0.43275400	-1.16970000
H	6.13537800	-1.51906100	-1.29829700
H	5.26631500	-0.08525400	-1.83776900
H	7.00380300	0.01096000	-1.50820800
C	7.33571400	2.12714800	3.00105600
H	8.15143200	1.56537500	3.47559700
H	7.79319700	2.84789800	2.31531700
H	6.82311500	2.68724900	3.78975700
H	-0.44818500	2.81686800	-3.80956900
H	-2.10591000	2.19965900	-3.61380800
H	2.10539400	-2.19894700	-3.61455700
H	0.44764000	-2.81611500	-3.81019200

{Ag[NHC^{Mes,iPr}]}$_2$, **2c$_c$**

Ag	1.86755900	0.93720100	-0.38986300
O	0.37118500	-4.40409000	-1.16575100
N	2.19703700	-2.14712500	-0.86002200
N	3.69845700	-1.41402900	0.50085500
C	2.64301000	-1.01355600	-0.26230300
C	3.90126300	-2.78518600	0.37909000
H	4.68612900	-3.29550800	0.91501100
C	2.95318500	-3.24625900	-0.47716200
H	2.69428900	-4.23775800	-0.81017100
C	1.05759400	-2.20933200	-1.79710900
H	0.64449500	-1.19754600	-1.80895200
C	0.00600800	-3.23002900	-1.25327500
O	-1.16625900	-2.77939700	-0.97633300
C	1.50592200	-2.59673500	-3.22576500
H	1.89139100	-3.62256900	-3.16620300
C	4.48980200	-0.54201900	1.33288200
C	5.62628200	0.07474500	0.78383600
C	4.10332600	-0.34453900	2.66863900
C	6.37900900	0.91030700	1.61431200
C	4.89116900	0.50076800	3.45620500
C	6.03036900	1.13535400	2.95051600
H	7.25914600	1.40017500	1.20375100
H	4.60263700	0.66953500	4.49121000
C	2.87137400	-1.00607800	3.23983200
H	1.96246800	-0.65843000	2.73467000
H	2.90115500	-2.09559100	3.12407600
H	2.77231100	-0.78117500	4.30550600
C	6.02088100	-0.14046100	-0.65825600
H	6.12553100	-1.20509500	-0.89710400
H	5.26784600	0.26749700	-1.34311500
H	6.97379600	0.35053600	-0.87519300
C	6.87997400	2.02052100	3.83243500
H	7.69309700	1.44890200	4.29967200

H	7.34037700	2.83207800	3.25941600
H	6.28928900	2.46683300	4.63888700
Ag	-1.86871900	-0.85025700	-0.55060400
O	-0.37424500	4.54483800	-0.33260700
N	-2.20008100	2.26952400	-0.44957900
N	-3.69855500	1.29591200	0.75465500
C	-2.64275100	1.04458000	-0.06904800
C	-3.90532800	2.66555000	0.88569500
H	-4.69096300	3.06706700	1.50634600
C	-2.95881700	3.27797300	0.12841800
H	-2.70233200	4.31431900	-0.01677500
C	-1.06402400	2.50762800	-1.36273900
H	-0.64932300	1.51742600	-1.56750000
C	-0.01019900	3.40818700	-0.64077800
O	1.16268000	2.91314900	-0.45807100
C	-1.52236400	3.15644900	-2.69019300
H	-1.92741800	4.14364400	-2.43349200
C	-4.48504500	0.28370600	1.41422200
C	-5.60931800	-0.24178500	0.75415000
C	-4.10740500	-0.13858800	2.69835500
C	-6.35684900	-1.21803300	1.41744900
C	-4.89155200	-1.11759400	3.31893900
C	-6.01591900	-1.66908700	2.69829700
H	-7.22736900	-1.63815200	0.91826800
H	-4.61060600	-1.45900300	4.31252900
C	-2.88862700	0.42594300	3.38964000
H	-1.96980200	0.17222900	2.84745000
H	-2.92496900	1.51925800	3.45639700
H	-2.80206200	0.02714100	4.40431200
C	-5.99791600	0.21904300	-0.63110400
H	-6.14081400	1.30518200	-0.67396300
H	-5.22419400	-0.03027000	-1.36694700
H	-6.92985400	-0.25748600	-0.94793800
C	-6.85877700	-2.71153400	3.39539100
H	-7.81246700	-2.28706300	3.73566400
H	-7.09738500	-3.54487100	2.72507500
H	-6.34663500	-3.11905900	4.27229300
C	2.60889700	-1.67370300	-3.76206100
H	2.26093400	-0.63421000	-3.82486100
H	3.50372700	-1.68959500	-3.13098300
H	2.90677300	-1.98453100	-4.77003100
C	0.29208900	-2.60463500	-4.16886200
H	-0.47887400	-3.30350000	-3.83075700
H	-0.15982300	-1.60622200	-4.23887300
H	0.59798000	-2.90131700	-5.17826000
C	-0.31302900	3.37241800	-3.61397400
H	0.43956900	4.01770800	-3.15116500
H	0.16583800	2.41768600	-3.86855600
H	-0.63168200	3.84325100	-4.55068400
C	-2.60930700	2.33363200	-3.39561400
H	-2.23909900	1.33594500	-3.66606500
H	-3.49870000	2.20215600	-2.77050400
H	-2.92236900	2.83216700	-4.32011700

{Ag[NHC^{Mes,iBu}]}$_2$, **2d$_c$**

Ag	1.88147200	0.30791300	1.14352100
O	0.53600400	1.98157300	-4.06380700
N	2.30656600	1.16015600	-1.84604800
N	3.70116300	-0.38098500	-1.27955000
C	2.69766200	0.40211300	-0.79210800
C	3.92494500	-0.11197900	-2.62618800
H	4.67606800	-0.63670300	-3.19551100
C	3.04394900	0.85818100	-2.98238500
H	2.82230200	1.32931000	-3.92607700
C	1.24586000	2.18880500	-1.79635500
H	0.81304700	2.09979400	-0.79922200
C	0.17637900	1.87099800	-2.89031000
O	-1.00157800	1.54976700	-2.48343900
C	1.85631800	3.58542300	-1.99870300
H	2.31924900	3.60513000	-2.99168700
C	4.42816500	-1.35950100	-0.51018100
C	5.58701100	-0.95934400	0.17648800
C	3.96029400	-2.68269400	-0.47782600
C	6.27621700	-1.92809100	0.91102800

C	4.68763200	-3.61321500	0.27203100
C	5.84488000	-3.25817400	0.97145600
H	7.17215600	-1.63314500	1.45284200
H	4.33524100	-4.64132400	0.31240800
C	2.70562000	-3.09485900	-1.21108300
H	1.82324000	-2.58249300	-0.80912500
H	2.75559000	-2.84950400	-2.27817000
H	2.54178100	-4.17217800	-1.11815200
C	6.07299900	0.47053700	0.13870100
H	6.24248100	0.81685600	-0.88759600
H	5.34215000	1.15290800	0.58845900
H	7.01236300	0.57232400	0.68930000
C	6.62675700	-4.28688000	1.75460900
H	7.49628100	-4.64079000	1.18486100
H	7.00352700	-3.87102100	2.69532200
H	6.01213800	-5.16062000	1.99268800
Ag	-1.69518300	0.55872700	-0.77222400
O	-0.40093900	-0.85434400	4.43054900
N	-2.18929500	-0.36053700	2.19464400
N	-3.36969800	-1.51945900	0.81350100
C	-2.47768700	-0.49125100	0.87522300
C	-3.62640000	-2.02518400	2.08388700
H	-4.30156900	-2.85117300	2.24350900
C	-2.87878600	-1.29646400	2.95240000
H	-2.71439400	-1.38254200	4.01332200
C	-1.24077300	0.62390700	2.75496200
H	-0.85233600	1.17039700	1.89502400
C	-0.08220700	-0.12930800	3.48774500
O	1.11492500	0.11763300	3.08286300
C	-1.92876000	1.58251800	3.74414400
H	-2.23886500	0.99443400	4.61575900
C	-3.95205500	-2.03165300	-0.40146700
C	-5.15265400	-1.47268000	-0.86998900
C	-3.30448900	-3.08179600	-1.07231500
C	-5.69903000	-1.99490900	-2.04588700
C	-3.89345100	-3.56781200	-2.24396200
C	-5.08781100	-3.04099500	-2.74595000
H	-6.62592500	-1.56987500	-2.42465200
H	-3.40192900	-4.37729200	-2.77878500
C	-2.00800800	-3.66372700	-0.56013100
H	-1.21072300	-2.91092600	-0.54691100
H	-2.10529600	-4.03910500	0.46514900
H	-1.68017600	-4.49247500	-1.19408900
C	-5.83100200	-0.33646800	-0.14132300
H	-6.02136000	-0.58241100	0.90983200
H	-5.21269900	0.56911400	-0.14979000
H	-6.78832700	-0.09446700	-0.61150200
C	-5.71869200	-3.60428400	-3.99798700
H	-6.47900500	-4.35781200	-3.75249200
H	-6.21373400	-2.82212600	-4.58309100
H	-4.97415600	-4.08726900	-4.63870300
H	2.65584200	3.72721400	-1.25662600
H	-1.15196800	2.27018800	4.10744300
C	-3.11955700	2.40001800	3.20768000
H	-3.85622800	1.69818100	2.78947800
C	-3.79646100	3.14946200	4.36667900
H	-4.66886000	3.71330300	4.01587500
H	-4.13313000	2.45843000	5.14859100
H	-3.10332000	3.86364800	4.82988600
C	-2.71399800	3.37952400	2.09632500
H	-2.31389100	2.86393900	1.21572500
H	-3.57559100	3.97025600	1.76308400
H	-1.94759800	4.08110600	2.45145700
C	0.85190100	4.75096000	-1.90269100
H	0.06223300	4.57216500	-2.64533700
C	1.55201000	6.06812900	-2.27192500
H	0.84551900	6.90623600	-2.25499600
H	2.35821100	6.29871100	-1.56274700
H	1.99119500	6.01901500	-3.27520600
C	0.19534500	4.85745500	-0.51765500
H	-0.40052400	3.97133000	-0.27421100
H	0.95167900	4.98541600	0.26881000
H	-0.47746300	5.72193300	-0.47390000
